# Identifying the effective behaviour change techniques in nutrition and physical activity interventions for the treatment of overweight/obesity in post-treatment breast cancer survivors: a systematic review

**DOI:** 10.1007/s10552-023-01707-w

**Published:** 2023-05-06

**Authors:** Maria Perperidi, Dimitra Saliari, Christos Christakis, Inge Huybrechts, Emmanouil Saloustros, Yannis Theodorakis, Odysseas Androutsos

**Affiliations:** 1 Laboratory of Clinical Nutrition and Dietetics, Department of Nutrition and Dietetics, School of Physical Education, Sport Science and Dietetics, University of Thessaly, 1C Argonafton, 42132 Trikala, Thessaly, Greece; 2 International Agency for Research on Cancer, World Health Organization, Lyon, France; 3 Department of Oncology, Medical School, University Hospital of Larissa, Larissa, Greece; 4 Department of Physical Education and Sport Science, School of Physical Education, Sport Science and Dietetics, University of Thessaly, Trikala, Thessaly, Greece

**Keywords:** Lifestyle interventions, Behaviour change theories, Behaviour change techniques, Breast cancer survivors, Obesity

## Abstract

**Purpose:**

Updated evidence for the treatment of obesity in cancer survivors includes behavioural lifestyle interventions underpinning at least one theoretical framework. The aim of this systematic review was to assess the effectiveness of theory-based lifestyle interventions for the treatment of overweight/obesity in breast cancer survivors and to report effective behavioural change techniques (BCTs) and components used in these interventions.

**Methods:**

Four databases were searched for RCTs published between database inception and July 2022. The search strategy included MeSH terms and text words, using the PICO-framework to guide the eligibility criteria. The PRISMA guidelines were followed. Risk-of-bias, TIDier Checklist for interventions’ content, and the extent of behaviour change theories and techniques application were assessed. To evaluate the effectiveness of interventions, trials were categorised as “very,” “quite,” or “non” promising according to their potential to reduce body weight, and BCTs promise ratios were calculated to assess the potential of BCTs within interventions to decrease body weight.

**Results:**

Eleven RCTs met the inclusion criteria. Seven trials were classified as “very”, three as “quite” and one study was “non” promising. Studies’ size, design, and intervention strategies varied greatly, but the weight-loss goal in all studies was ≥ 5% of the initial body weight through a 500–1000 kcal/day energy deficit and a gradually increased exercise goal of ≥ 30 min/day. Social Cognitive Theory was the most commonly used theory (*n* = 10). BCTs ranged from 10 to 23 in the interventions, but all trials included behaviour goal setting, self-monitoring, instructions on the behaviour, and credible source. The risk-of-bias was “moderate” in eight studies and “high” in three.

**Conclusion:**

The present systematic review identified the components of theory-based nutrition and physical activity behaviour change interventions that may be beneficial for the treatment of overweight/obesity in breast cancer survivors. The strategies mentioned, in addition to reported behavioural models and BCTs, should be considered when developing weight-loss interventions for breast cancer survivors.

**Supplementary Information:**

The online version contains supplementary material available at 10.1007/s10552-023-01707-w.

## Introduction

Obesity has been suggested as an independent risk factor for breast cancer prognosis [1, 2]. Greater body mass index (BMI) and adiposity are linked to adverse outcome in women with breast cancer, including increased risk of recurrence and mortality, in addition to poorer quality of life and increased risk of developing co-morbidities, such as type 2 diabetes, hypertension, and cardiovascular disease [2–6]. However, observational studies have shown that lifestyle modification, especially the improvement of nutrition and physical activity, would potentially benefit breast cancer survival along with improvements in metabolic parameters and reduction in the risk of co-morbidities [3, 6].

Patients after cancer diagnosis seem to have higher level of motivation to change their lifestyle behaviours than they had before cancer diagnosis [7–9]. Interventions that promote healthy nutrition and physical activity have been proposed as an effective approach to support cancer survivors in lifestyle modification and weight loss [10, 11]. Current guidelines suggest that lifestyle modification could be successfully implemented through behavioural interventions [11, 12], especially when these are designed according to theoretical frameworks [13, 14]. Over the past years, many theories and models have been described in the literature, with Cognitive Behavioural Theory [15], Social Cognitive Theory [16] and the Transtheoretical Model [17] being the most commonly used theories in dietetic practice. Theory-based interventions facilitate an understanding of mechanisms of behaviour change providing the basis for developing more effective interventions [18]. This can be addressed by identifying the factors that influence behaviour called determinants, and select the appropriate behaviour change techniques to target behaviour [19]. Recent studies indicate that interventions delivered by credentialed healthcare professionals who are using behaviour change techniques may be more effective in improving patient health outcomes than dietary interventions which are not developed based on behavioural theories [13]. Effective behavioural change techniques in interventions for cancer survivors include goal setting, problem solving and social support [9].

Τhe implementation of lifestyle interventions in breast cancer survivors, however, is still limited [2, 12]. The majority of previous literature reviews focusses on the impact of body weight loss on breast cancer survival, without taking into account the methods used to achieve lifestyle modification or describing the treatment mechanisms and/or the behaviour change techniques used to successfully treat overweight/obesity in breast cancer survivors [1, 2, 4, 5]. Although there are few reviews focussing on lifestyle interventions in cancer survivors [2, 12, 20], reviews addressing breast cancer are scarce. Some reviews examine either the dietary [7, 21, 22], or the physical activity [21, 23–25] interventions, but not the combination of both lifestyle behaviours on weight control. A recently published review [26] focussed on weight-loss interventions in breast cancer survivors targeting different behaviours, such as ‘diet’, ‘exercise’, ‘psychosocial support’ and/or their combinations. Although all the interventions included resulted in a reduction in BMI, the subgroup analyses showed that interventions that combined multiple factors, such as ‘diet and exercise’ or ‘diet, exercise and psychosocial support’, led to greater improvements in anthropometric indices and weight status compared to ‘diet’-only interventions. Despite the fact that this systematic review and meta-analysis rigorously examines various elements and/or their combinations on weight loss in breast cancer survivors, the study does not focus on the interventions’ constructs and BCTs, which could facilitate behaviours modification, since this was not within their scope. Finally, even less reviews have examined the effectiveness of weight-loss intervention in breast cancer survivors based on their theoretical framework [22, 24, 25]. Given that multifactorial interventions may be more effective in managing body weight, identifying the elements and BCTs used in the most effective interventions may result in the greatest benefit in this population.

The aim of this systematic review was to assess the effectiveness of theory-based lifestyle interventions for the treatment of overweight/obesity in breast cancer survivors and to report effective behavioural models and strategies used in these interventions.

## Methods

This systematic review was conducted according to the Preferred Reporting Items for Systematic Reviews and Meta-Analyses (PRISMA) guidelines [27], and a PRISMA checklist is provided.

It has also been registered with the International Prospective Register of Systematic Reviews, PROSPERO (CRD42021252827).

### Inclusion/exclusion criteria

The targeting population was female, adult, breast cancer survivors with BMI ≥ 25 kg/m^2^ and without any active cancer therapy or any ongoing treatment, except of hormonal or immune therapy. The eligible interventions included both dietary and physical activity components targeting overweight/obesity with the use of at least one behaviour change theory or model. Randomized Control Trials (RCTs) with at least one control or comparison group of weight-loss interventions were eligible for inclusion. Original articles/studies published in English language, between database inception and July 2022 were included.

Animal studies, pharmacological studies, non RCT studies, RCTs not published in English, trials with breast cancer survivors with active cancer, trials without a specific theoretical framework such as lifestyle interventions with behavioral counselling in general, conference proceedings, letters, reviews or meta-analyses were excluded.

In this review, breast cancer survivors were defined as women who have received a diagnosis of breast cancer and having completed overall treatment, including surgery, chemotherapy, radiotherapy and other cancer treatments, excluding hormone or immune therapy.

### Search methods

A structured search was conducted, focussing on the 4 following databases: PubMed/Medline, Scopus, TripDatabase and Central/Cochrane. The search strategy included Medical Subject Headings (MeSH) and text words. These terms were related to behavioural interventions, breast cancer survivors, obesity/overweight, and weight loss/BMI reduction, using the PICO (Population, Intervention, Comparison, Outcome) framework to guide the eligibility criteria. Consistently, the following terms were combined: (Breast Neoplasm OR breast cancer OR breast cancer survivors) AND (lifestyle intervention OR behavioural intervention OR behavioural intervention OR theory-based intervention OR theoretical framework) AND (obesity OR overweight) AND (weight loss OR weight-loss OR weight change OR weight management OR BMI reduction). No date, country or origin/ethnicity restrictions were applied. Publications were imported in Endnote, where data were checked, duplicates were removed, and the title and the abstract were screened. Additionally, reference lists of RCT studies were cross-matched and forward citation searching was conducted to detect additional studies that met the inclusion criteria. Moreover, the International Trials Registry was explored for ongoing trials. After the title screening, the remaining publications (*n* = 280) were uploaded in the Rayyan application [28] to complete the abstract screening process and the full-text review.

The research in the databases and the screening of the publications were independently implemented by two reviewers (M.P. and D.S.). Any disagreements during the screening process were discussed between M.P. and D.S and resolved through consultation with O.A.

The full search strategy is detailed in supplemental file 1.

### Data extraction

Data on study characteristics (author, publication year, title, study design and duration), participants’ characteristics (age, BMI), cancer site, study groups, intervention duration, lifestyle modifications, behavioural change model and weight change outcome were extracted. Information was collected from both the original articles (the included publications) along with any protocol/study design, previous or subsequent publication of each study. Data were checked by three reviewers (M.P., D.S., C.C.).

Interventions’ description was recorded by M.P. using the TIDier (Template for Intervention Description and Replication) Checklist, including 12 categories “brief name”, “why”, “what (materials)”, “what (procedures)”, “who provided”, “how”, “where”, “when and how much”, “tailoring”, “modifications”, “how well (planned)” and “how well (actual)” [29]. Total set of TIDier Checklist is included in supplemental file 2.

The Theory Coding Scheme [30] was used to evaluate the way in which behavioural theory has been applied within interventions. The final Theory Coding Scheme comprises 19 items, of which items 1–11 assess how theory and targeted constructs were used to developing the intervention, while items 12–19 evaluate methodological issues concerning the use of theory in the basis of the study outcomes. Provided that one of the aims in this systematic review was to appraise the application of theory to interventions, we decided to code items 1–11 and exclude items 12–19. The included studies were examined for the use of their theoretical framework only by one reviewer (M.P.) according to the main publication about the development of Coding Theory [30] and definitions related to theoretical constructs [18, 19, 31–33]. To facilitate the coding, a record was created between the targeted theoretical constructs and the intervention techniques of each trial, and is available in supplemental file 2, along with the results of the Theory Coding Scheme.

The Behaviour Change Technique Taxonomy [34] was used to identify the behaviour change techniques (BCTs) in the interventions. This taxonomy consists of 93 hierarchically clustered techniques which are utilised for specifying the active elements of behavioural change interventions. Each study was independently coded by two reviewers (M.P. and D.S.) and the disagreements were discussed and resolved through the third reviewer (C.C). The three reviewers (M.P., D.S. and C.C.) totally agreed to the final selection of BCTs for all the studies and were assisted by the definitions given from the main publication and supplementary material of the taxonomy along with the theoretical understanding of intervention evaluations [18, 19]. The BCTs taxonomy mapping of the studies is presented in supplemental file 2.

### Data synthesis

Given the wide variety of the interventions’ content and design as well as the aims of this review, a meta-analysis was not appropriate. As a result, a narrative synthesis of the content and the promise of the interventions (based on criteria used in previous reviews by Gardner et al. and Moore et al.) was used as guidance for the development of more effective future interventions [35, 36].

Interventions’ content was assesses using the TIDier (Template for Intervention Description and Replication) Checklist [29]. Total set of TIDier Checklist is included in supplemental file 2.

Interventions’ promise classification system as described by Gardner [35] was used to assess the effectiveness of each study. According to this method, interventions were grouped into three categories of “promise” depending on their potential post-intervention reductions in participants’ body weight (statistically significant within and/or between group). Interventions were considered “very promising” if there were statistically significant reductions in participants’ body weight within the intervention group, and this reduction was greater than observed in at least one comparator arm (control group or at least one other intervention group). Interventions were considered “quite promising” if there were either statistically significant reductions in participants’ body weight within the intervention group, or reduction in at least one comparator arm. Interventions were considered “non-promising” if no statistically significant decreases were found neither within intervention arm nor between study arms. This classification ensured that studies with the strongest evidence of their efficacy were distinguished from those with weaker evidence. Interventions’ promise was estimated independently by two reviewers (M.P., C.C.).

The potential of BCTs within interventions for facilitating weight loss was calculated with a “promise ratio” for each BCT. The “promise ratio” of a BCT is defined as the ratio of the number of “very promising” and the number of “quite promising” interventions in which this BCT was present, divided by the number of “non-promising” interventions of which this BCT was a component. BCTs were considered as promising if there were featured in at least twice as many “promising” (very and quite) as “non-promising” interventions (promise ratio ≥ 2). BCTs found in two or more “promising” interventions but in none “non-promising” intervention were reported as the number of “promising” interventions in which a BCT featured and not as a ratio. A promise ratio was not calculated if only appeared in non-promising interventions or only appeared once. The higher the ratio, the more promising the BCT.

### Risk of bias assessment

The revised version of Cochrane risk-of-bias tool for randomized trials (RoB 2) [37] was implemented for the included studies, examining the following five bias domains: randomization process; deviations from intended interventions; missing outcome data; measurement of the outcome; and selection of reported results. The robvis tool was used to visualize risk of bias assessment [38].

Each study was assessed independently by two reviewers (M.P. and D.S.) using the up-to-date information from the developers on RoB 2 such as the full guidance document and the key Cochrane resources for using RoB 2. Any discrepancies between them were resolved through discussion, and the final decision was made with their mutual consent. The overall risk of bias were defined as “low” or “high” or expressed as “some concerns” according to the assessment technique set out in the aforementioned tool and based on these criteria each study was rated as either “Low risk of bias” (for all domains), “Some concerns” (at least one domain raised some concerns but none high risk at any domain) or “High risk of bias” (at least one domain with high risk of bias or some concerns in multiple domains).

### Inter-rater agreement

Inter-rater agreement was calculated for BCTs taxonomy, risk of bias assessment, and interventions’ promise classification, using percentage agreement and kappa (κ) (0–0.20 = slight agreement, 0.20–0.40 = fair agreement, 0.40–0.60 = moderate agreement, 0.60–0.80 = substantial agreement, and > 0.80 = nearly perfect agreement) [39].

## Results

### Search results

**Fig. 1:**
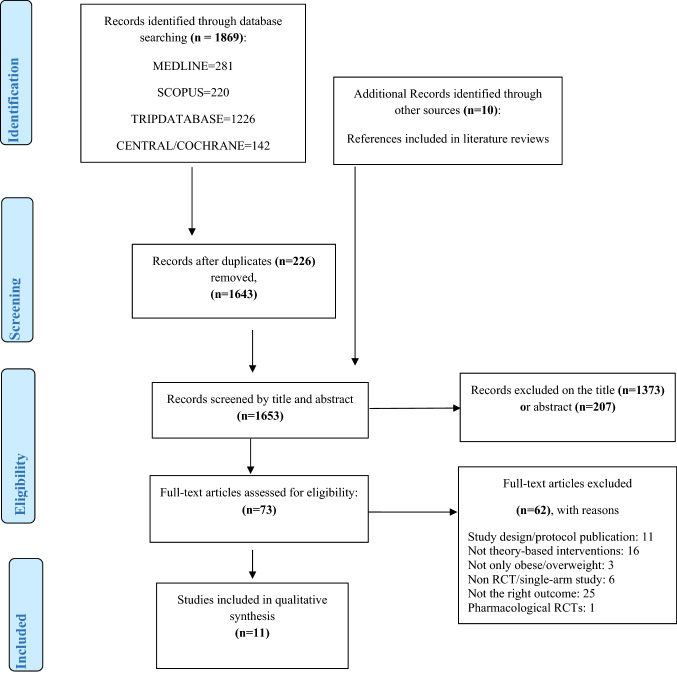
Preferred reporting items for systematic reviews and meta-analyses (PRISMA) flow diagram of the literature search and filtering results

Database searches yielded a total of 1869 articles from Medline (*n* = 281), Scopus (*n* = 220), Tripdatabase (*n* = 1226), and Central/Cochrane (*n* = 142). Screening the references list, ten additional articles were found. After duplicates were removed, a total of 1643 articles were screened by title and abstract, with 73 articles being selected for full-text screening. Eleven RCT studies were included in this systematic review, meeting the inclusion criteria [40–50]. The remaining 62 articles were excluded for a variety of reasons, including study design publication (*n* = 11), lack of theory-based interventions (*n* = 16), participants’ BMI < 25 kg/m^2^ (*n* = 3), non-RCT studies (*n* = 6), not weight loss as an outcome (*n* = 25), and pharmacological RCT (*n* = 1). The search results and selection process have been presented in the PRISMA (Preferred Reporting Items for Systematic Reviews and Meta-Analyses) flow diagram (Fig. 1). The full search strategy and the excluded studies are detailed in supplemental file 1.

### Studies’ characteristics and content

Interventions differed greatly regarding the number of samples, with the ENERGY trial [40] including the most participants (*n* = 692) and the Stepping Stone study [44] including the fewest. Three studies [46, 49, 50] included approximately 300 participants, while the remaining six [41–43, 45, 47, 48] had less than 100 samples.

The main characteristics (sample, design, intervention, lifestyle modification goals, outcome goal) of the eleven studies are summarized in Table 1. For the extraction of the results, any protocol [51–54], and any previous or subsequent publication [55–58] of each study was taken into account.

**Table 1:** Characteristics of the studies (design, intervention and goals)

Studies	Sample	Design	Intervention	Dietary goal	Physical activity goal	Weight loss goal
Rock, 2015 (ENERGY trial) [40]	*N* = 692	2- arm: a group-based behavioural intervention & telephone counselling & tailored newsletters OR a less intensive control intervention	6 months intensive phase: 4 months, 1 h weekly group sessions (av. 15 women/group) Next 2 months: 1 h group sessions every other week 6–12 months, monthly meetings. Every session followed by brief (10–15 min) personalised guidance delivered by tel and/or email 6–24 months: tailored newsletters	500–1000 kcal deficit for a WL of 1–2 pounds/week by increasing high-fibre vegetables, whole grains & fruits	step-wise increase in time & intensity regular planned aerobic exercise, increased PA in the lifestyle & strength training Long-term goal: 60 min/day of moderate intensity purposeful exercise, 10,000 steps per day & 2–3 times/week strength training at home or an exercise facility	At least 7% of BW at 2 years
Demark-Wahnefried, 2014 (DAMES trial) [41]	*N* = 68 dyads (breast cancer survivors & their daughters)	3- arm: a tailored diet & exercise intervention delivered individually to mothers & daughters (individual) OR emphasised the mother-daughter bond (team) OR control group	Individual arm: individual weight goals plus tailored feedback about their nutrition vs national guidelines & 6 subsequent newsletters Team arm: Same material to those in the individual arm but mother & daughter received the material together	Lower calorie substitutes or provided guidance on portion control	Both interventions groups: 150 min per week of aerobic exercise and twice-weekly strength training	Not specified, personalised
Mefferd, 2006 (HWM study) [42]	*N* = 85	2- arm: intervention OR wait-list group	12 months duration: 16 weeks of weekly closed groups sessions followed by once-monthly session till 12 months	500–1000 kcal deficit for a WL by increasing high-fibre vegetables, whole grains & fruits	Regular aerobic exercise with a step-wise increase in time and intensity Long-term goal of 1 h/day of moderate to vigorous PA & 2–3 times/week muscle strengthening	Not specified
Djuric, 2002 [43]	*N* = 48	4- arm: control/Weight Watchers (WW)/individualised counselling/WW plus individualised counselling	Individualised arm: weekly sessions for the first 3 months, biweekly for 3–6 months & monthly thereafter, plus monthly packet of written information Comprehensive arm: individualised counselling plus weekly WW meetings without the monthly dietitian-led meetings	500–1000 kcal deficit for a WL of 1–2 pounds/week, by decreasing energy & fat & increasing fibres. At least 5 servings/day fruits & vegetables. Target fat at 20–25% & protein up to 20% of total energy	30–45 min/day moderate activity most days of the week	10% at 6 months
Sheppard, 2016 (Stepping Stone study) [44]	*N* = 31	2- arm: intervention VS usual care (control)	12 weeks intervention: once biweekly a 90-min group sessions (30 min PA & 60 min education sessions) co-led by an exercise physiologist & a nutritionist, plus individual telephone (15 min) coaching sessions every other week	1 pound of WL per week. Dietary recommendations: > 5 fruits and vegetables/day and < 35% kcal from total fat	Moderate intensity exercise of > 30 min/day, ≥ 5 days/week. PA goal of 10,000 steps/day for 12 weeks	5% in 12 weeks
Harrigan, 2016 (LEAN study) [45]	*N* = 100	3- arm: in-person counselling OR telephone counselling OR usual care (control)	Same intervention for both intervention groups: 6 months: 11 sessions (30-min counselling), (once weekly the first month, once biweekly the second & third month and once monthly for the months 4,5,6), by a RD specialised in oncology nutrition and trained in exercise physiology & behaviour modification counselling	500 kcal energy deficit based on a dietary fat goal of < 25% of total energy intake, a plant-based diet reducing sugars and increasing fibre	Home based PA with a goal of 150 min per week moderate-intensity activity, such as brisk walking, with a daily target of 10,000 steps	10% at 6 months
Stolley, 2017 (Moving Forward trial) [46]	*N* = 246	2- arm: 6-months Moving Forward Interventionist—Guided program (MFG) OR the Moving Forward Self—Guided program (SG)	MFG included twice—weekly (for 26 weeks) in-person classes with supervised exercise & text messaging targeting self-efficacy	500 kcal deficit by increasing fruit & vegetable consumption	Minimum ≥ 150 min per week	5% at 6 months
Santa-Maria, 2020 (POWER-remote trial) [47]	*N* = 87	2- arm: POWER-remote VS self-directed (control)	12-month behavioural weight loss intervention (telephone-based coaching & use of a web-based self-monitoring and learning platform). A total of 21 phone calls: weekly for 3 months & monthly for 9 months (20 min calls per session)	1200–2200 kcal/day energy intake depending on the body weight through DASH dietary pattern: 7–12 servings of fruits/vegetables, 2–3 servings of low fat dairy, reduced sodium & ≤ 25% of calories from fat	Built to ≥ 300 min/week of moderate intensity PA in bouts ≥ 10 min in length	At least 5% at 6 months
Reeves, 2017 (Living Well after Breast Cancer trial) [48]	*N* = 90	2- arm: weight loss intervention (diet & PA) VS usual care (control)	12 months phone-delivered intervention 6 months initial phase: A total of 16 phone calls (weekly for 6 weeks & 10 fortnightly calls) 6 months extended care phase: 6 monthly calls	2000 kj (≈ 500 kcal) daily energy deficit aiming to ≤ 30% total fat, < 7% saturated fat, 5 servings/day vegetables, 2 servings/day fruits, limit alcohol intake & portion control	Gradually increased moderate intensity planned PA to at least 30-min/day (≥ 210 min/week). To increase incidental activity & to reduce sedentary behaviour	5–10% at 6 months
Schmitz, 2019 (WISER Survivor trial) [49]	*N* = 351	4- arm: Home-Based Exercise Intervention/Weight Loss Intervention/ Combined Intervention & Control	12 months (52 weeks) home-based exercise program of strength training twice/week & 180 min/week walking along with 24 weeks nutritional counseling group sessions	Guidelines from ACS along with a meal replacement program (Nutrisystem) & 7 servings fruits and vegetables daily	Twice per week resistance exercise per 90-min class along with aerobic activity to 180 min per week	10% at 6 months
Goodwin, 2014 (LISA trial) [50]	*N* = 338	2- arm: individual lifestyle intervention (LI) VS mail-based education intervention (control)	24 months telephone-based intervention: 6 months of the intensive (weekly for 4 weeks) & consolidation phase (fortnightly for 2–6 months) & 18 months of the maintenance phase (every 2 months for 7–12 and every 3 months for 12–24 months)	500–1000 kcal deficit for a WL of 1–2 lbs/week, by decreasing fat to 20% of total intake & increasing fruits, vegetables & fibres	A gradual increase in moderate-intensity aerobic physical activity (walking for the majority of participants) to 150 to 200 min per week	10% at 6 months

*PA* physical activity, *WL* weight loss, *BMI* body mass index, *RD* registered dietitian, *ACS* American cancer society

Interventions’ content were recorded using TIDier Checklist in Table 2.

**Table 2:** Interventions’ content by TIDier checklist

Studies	12 items TIDier
Rock [40]	Brief Name: Exercise and Nutrition to Enhance Recovery and Good Health for you (ENERGY) Trial WHY: To determine whether a behavioural WL intervention emphasizing increased PA and tailored to BCS would result in greater WL in the IG compared with a CG assigned to a less intensive intervention Theory: Social Cognitive Theory and Cognitive—Behavioural treatment of obesity, with Motivational Interviewing WHAT: Materials: Tailored print materials from previous trials with web-based resources, digital videos, pedometers and weight records. Procedures: The goal was a modest WL of at least 7% BW through behavioural goals such as reduced energy intake and increased physical activity, by personalized guidance WHO: leaders, who had backgrounds in dietetics, psychology and/or exercise physiology HOW: face-to-face group sessions, emails, telephone, newsletters WHERE: USA (University of California, San Diego [UCSD]; University of Colorado Denver; University of Alabama at Birmingham; and Washington University in St. Louis [WUSTL]) WHEN AND HOW MUCH: 6 months intensive phase: 4 months, 1 h weekly group sessions and next 2 months, 1 h group sessions every other week. 6–12 months: monthly meetings, followed by brief (10–15 min) personalised guidance delivered by tel and/or email. 6–24 months: tailored newsletters. Physical Activity: Step-wise increase to 60 min/day of moderate intensity purposeful exercise, 10,000 steps per day & 2–3 times/week strength training at home or an exercise facility TAILORING: individualize the feedback, goal setting, planning and follow-through for the behavioural goals, diet and PA MODIFICATIONS: None described HOW WELL (actual & planned): in appendix
Demark-Wahnefried [41]	Brief Name: Daughters and Mothers Against Breast Cancer (DAMES) trial WHY: endeavored to capitalize on the mother-daughter bond and the teachable moment created by a cancer diagnosis to promote weight loss in overweight or obese women recently diagnosed with breast cancer and their overweight or obese daughters Theory: Social Cognitive Theory & Transtheoretical model of behaviour change, plus concepts of independence theory and the theory of communal coping WHAT: Materials: workbook that was personalized with reinforced goals proposed by the ACS and the US dietary guidelines, 6 newsletters, logbooks, reference manuals, web sites, portion control tableware, iPods, shoes chips. Procedures: promoted portion control and diets high in nutrients and low in energy as well as 150 min per week of aerobic exercise and twice-weekly strength training WHO: Not described HOW: emails and newsletters WHERE: USA, Puerto Pico or Guam WHEN AND HOW MUCH: 6 months intervention and 6 months follow-up phase TAILORING: interventions differed with respect to tailoring MODIFICATIONS: None described HOW WELL (actual & planned): in appendix
Mefferd [42]	Brief Name: The Healthy Weight Management (HWM) Study WHY: The intervention incorporated CBT, emphasizing PA, diet modification to facilitate a modest reduction in energy intake, and strategies to improve body image and self-acceptance Theory: Cognitive Behavioural Therapy WHAT: Materials: food diaries, exercise logs, pedometers and intervention material. Procedures: 500–1000 kcal deficit for a WL by increasing high-fibre vegetables, whole grains & fruits and regular aerobic exercise with a step-wise increase in time and intensity (1 h/day of moderate to vigorous PA & 2–3 times/week muscle strengthening) WHO: trained investigators and research staff HOW: in-person group sessions and telephone contacts WHERE: USA (San Diego) WHEN AND HOW MUCH: 16 weeks of weekly closed groups sessions followed by once-monthly session till 12 months TAILORING: Individualized telephone counseling to individualize goal setting and assess progress MODIFICATIONS: None described HOW WELL (planned): Not described HOW WELL (actual): in appendix
Djuric [43]	Brief Name: Randomized pilot study tested an individualized approach toward weight loss in obese Breast cancer survivors WHY: individualized counseling methods typically have not been used in WL research studies but this approach is sensitive to the needs and abilities of each individual Theory: Social Cognitive Theory WHAT: Materials: A monthly packet of written information on various WL topics (environmental control, serving-size control, exercise, motivation, goal setting, holiday eating, seasonal foods), pedometers, exercise and dietary logs. Procedures: 10% WL of BW through 500–1000 kcal deficit for a WL of 1–2 pounds/week, by decreasing energy & fat & increasing fibres and at least 5 servings/day fruits & vegetables plus 30–45 min/day moderate activity most days of the week WHO: Registered dietitian HOW: in-person group meetings, telephone individual counselling and emails WHERE: USA WHEN AND HOW MUCH: weekly sessions for the first 3 months, biweekly for 3–6 months & monthly thereafter TAILORING: depending on individual needs MODIFICATIONS: None described HOW WELL (planned): Not described HOW WELL (actual): in appendix
Sheppard [44]	Brief Name: Stepping Stone (Survivors Taking on Nutrition and Exercise) study WHY: Studies with white survivors suggest that interventions are more effective when they are multifaceted, personalized, teach behavioural skills, provide social support, and increase self- efficacy. This is also likely true for black survivors, but documentation of successful strategies for them are lacking Theory: Social Cognitive Theory & Theory of Planned Behaviour, with Motivational Interviewing WHAT: Materials: pedometers, notebooks, tools to monitor and track their daily food intake, and binders to store resources and session materials. Procedures: WL of at least 5% BW in 12 weeks, through 1 pound of WL per week, > 5 fruits and vegetables/day and < 35% kcal from total fat and moderate intensity exercise of > 30 min/day, ≥ 5 days/week, and 10,000 steps/day WHO: exercise physiologist, nutritionist and trained survivor coach HOW: in-person group sessions, plus individual telephone coaching sessions WHERE: USA WHEN AND HOW MUCH: 12 weeks intervention: once biweekly a 90-min group sessions (30 min PA & 60 min education sessions), plus individual telephone (15 min) coaching sessions every other week TAILORING: individualized sessions were tailored to baseline intentions, attitudes, and subjective norms MODIFICATIONS: None described HOW WELL (actual & planned): in appendix
Harrigan [45]	Brief Name: The Lifestyle, Exercise, and Nutrition (LEAN) Study WHY: Telephone-based weight loss counselling may be a viable time-effective alternative to in-person visits Theory: Social Cognitive Theory WHAT: Materials: 11-chapter LEAN book, daily record of all food and beverage intake, minutes of physical activity, and pedometer steps in the LEAN Journal and weighed themselves once per week with a scale, and recorded their weight in the LEAN Journal. Procedures: WL of at least 10% BW in 6 months, through 500 kcal energy deficit based on a plant-based diet reducing sugars and increasing fibre and home-based PA with a goal of 150 min per week moderate-intensity activity, such as brisk walking, with a daily target of 10,000 steps WHO: RD specialised in oncology nutrition and trained in exercise physiology & behaviour modification counselling HOW: either in-person or telephone individual sessions WHERE: Yale, USA (p.670) WHEN AND HOW MUCH: 6 months, 11 sessions (30-min counselling), (once weekly the first month, once biweekly the second & third month and once monthly for the months 4,5,6) TAILORING: participants received individualised counselling sessions MODIFICATIONS: None described HOW WELL (actual & planned): in appendix
Stolley [46]	Brief Name: Moving Forward trial, a WL intervention for African-American BCS on weight, body composition and behaviour WHY: Body composition and biological data will enhance the understanding of how WL may impact BC recurrence risk and overall health risk among African-American women Theory: Social Cognitive Theory and Socio-Ecological Model, with Motivational Interviewing WHAT: Materials: classes with specific topics of diet and exercise, weight, food and activity records, program binder with handouts, recipes, and other supportive materials. Procedures: WL of at least 5% BW in 6 months, through 500 kcal deficit by increasing fruit & vegetable consumption and PA ≥ 150 min per week WHO: a community dietitian, a community cancer exercise instructor, and a health psychologist HOW: in-person group sessions, text messages through a software application, mytapp and newsletters WHERE: USA, Chicago area WHEN AND HOW MUCH: twice—weekly (for 26 weeks) in-person classes with supervised exercise & text messaging TAILORING: Not described MODIFICATIONS: Intervention goals change briefly HOW WELL (planned): Not described HOW WELL (actual): in appendix
Santa-Maria [47]	Brief Name: POWER-remote trial, Practice-based Opportunities for Weight Reduction for breast cancer survivors WHY: BC Patients with obesity experience inferior outcomes, biologically related to metabolic and inflammatory pathways, and other molecular changes. WL may be associated with decreases in leptin and other inflammatory markers, which may have antioncogenic effects Theory: Social Cognitive Theory with Motivational Interviewing WHAT: Materials: educational materials included oncology-relevant information such as lymphedema prevention exercises and general information about BC, web-based resources with objectives, educational content, quizzes, and supporting worksheet and self-monitoring tools and graphs (weight, minutes of exercise/day, calories consumed/day). Procedures: WL of at least 5% BW in 6 months, through 1200–2200 kcal/day energy intake depending on BW based on DASH dietary pattern: 7–12 servings of fruits/vegetables, 2–3 servings of low fat dairy, reduced sodium & ≤ 25% of calories from fat and built up to ≥ 300 min/week of moderate intensity PA in bouts ≥ 10 min in length WHO: health coaches with a background in delivering weight loss interventions HOW: telephone-based behavioural WL coaching and use of a web-based self-monitoring and learning platform WHERE: USA WHEN AND HOW MUCH: 12-months (telephone-based coaching & use of a web-based self-monitoring and learning platform). A total of 21 phone calls: weekly for 3 months & monthly for 9 months (20 min calls per session) TAILORING: Individually tailored MODIFICATIONS: None described HOW WELL (actual & planned): in appendix
Reeves [48]	Brief Name: Living Well after Breast Cancer Pilot Trial WHY: Comparisons of interventions against usual care are still warranted, particularly when examining patient-reported outcomes and treatment-related side-effects, as these may naturally improve over time following treatment completion Theory: Social Cognitive Theory with Motivational Interviewing WHAT: Materials: a detailed workbook, self-monitoring diary, digital scales, pedometer, calorie-counter book, food model booklet. Procedures: WL of 5–10% BW in 6 months, through 2000 kj ( ≈ 500 kcal) daily energy deficit aiming to ≤ 30% total fat, < 7% saturated fat, 5 servings/day vegetables, 2 servings/day fruits, limit alcohol intake & portion control and gradually increased moderate intensity planned PA to at least 30-min/day (≥ 210 min/week) and 10,000 steps/daily WHO: lifestyle coaches, who were accredited practicing dietitians trained in exercise promotion and motivational interviewing HOW: individual telephone sessions, optional supportive text messages and newsletters WHERE: Australia, within 50 km of the state capital, Brisbane WHEN AND HOW MUCH: 12 months: 6 months initial phase: A total of 16 phone calls (weekly for 6 weeks & 10 fortnightly calls) and 6 months extended care phase: 6 monthly calls (p5, 2016, study TAILORING: tailored to the participant’s preferences and individualised guidance MODIFICATIONS: None described HOW WELL (actual & planned): in appendix
Schmitz [49]	Brief Name: Women in Steady Exercise Research (WISER) Survivor clinical trial WHY: to test the effects of exercise and/or WL on lymphedema, biomarkers for recurrence and quality of life. The hypothesis is that exercise and weight loss will affect these outcomes, but that the combined effect will be larger Theory: Social Cognitive Theory and Behavioural Self-Management Theory, with Motivational Interviewing WHAT: Materials: exercise and food logs using an electronic food diary accessible through the WISER Survivor website. Procedures: WL of 10% BW in 6 months, based on the guidelines from ACS along with a meal replacement program & 7 servings fruits and vegetables daily, plus twice per week resistance exercise per 90-min class along with aerobic activity to 180 min per week WHO: registered dietitians experienced with the NutriSystem program and exercise by certified exercise instructors HOW: in-person group meetings along with telephone individual counselling WHERE: USA, Pennsylvania WHEN AND HOW MUCH: 12 months (52 weeks) home-based exercise program of strength training twice/week & 180 min/week walking along with 24 weeks nutritional counselling group sessions TAILORING: will develop a tailored diet that meets the same calorie control goals and the WL intervention was tailored to the needs of BCS MODIFICATIONS: None described HOW WELL (actual & planned): in appendix
Goodwin [50]	Brief Name: Lifestyle Intervention in Adjuvant Treatment of Early Breast Cancer (LISA) trial WHY: Obesity is a complex physiologic state associated with insulin resistance, higher levels of circulating insulin, an altered adipocytokine profile (increased leptin, decreased adiponectin), and generalized inflammation. WL may improve BC outcomes Theory: Social Cognitive Theory with Motivational Interviewing WHAT: Materials: Detailed patient workbook which focus on weight control through healthy diet and exercise with logs and pedometer. Procedures: WL of 10% BW in 6 months, through 500–1000 kcal deficit for a WL of 1–2 lbs/week, by decreasing fat to 20% of total intake & increasing fruits, vegetables & fibres and a gradual increase in moderate-intensity aerobic physical activity (walking for the majority of participants) to 150–200 min per week WHO: trained lifestyle coaches HOW: individual telephone sessions and newsletters WHERE: Canada, Ontario Clinical Oncology Group WHEN AND HOW MUCH: 24 months telephone-based intervention: 6 months of the intensive (weekly for 4 weeks) & consolidation phase (fortnightly for 2–6 months) & 18 months of the maintenance phase (every 2 months for 7–12 and every 3 months for 12–24 months) TAILORING: Lifestyle coaches individualized the intervention as necessary MODIFICATIONS: Patients with N3 tumour characteristic were allowed in initial protocol, amended June 2008, when 49 patients had been accrued HOW WELL (actual & planned): in appendix

*BCS* breast cancer survivors, *PA* physical activity, *WL* weight loss, *IG* intervention group, *CG* control group, *BW* body weight, *ACS* American cancer society

#### Interventions design and strategies

Interventions varied in their study design, but all of them had a control group. Specifically, most of them, seven of the eleven studies, had a 2-arm design [40, 42, 44, 46–48, 50] which means an intervention group and a control or a comparison group. Two of the studies had a 3-arm design [41, 45]. Specifically, in Dames trial [41]), a study involving 68 dyads of mothers breast cancer survivors and their daughters, the three groups included were the individual, the team (mothers and daughters) and the control group. The intervention given was the same for the individual and the team group, but in the first group a tailored diet and exercise were delivered individually to mothers and daughters while in the second group the intervention was given as a team, emphasising to the mother-daughter bond. In the LEAN study [45], 100 breast cancer survivors were divided into three groups: an in-person counselling group, a telephone counselling group and the control group. The intervention was the same for the counselling groups but it was given in a different manner, in-person or telephone-delivered. Finally, two studies had a 4-arm design [43, 49]. In the trial of Djuric et al., 48 breast cancer survivors were randomly assigned to one of the four groups: control, the commercial Weight Watchers program (WW), individualised counselling or a combination of the WW program and the individualised counselling. In the Wiser trial of Schmitz et al., 351 breast cancers survivors were randomized to either control or home-based exercise intervention or weight-loss intervention or the combined intervention (exercise and weight-loss). Every of these interventions was different to each group, as described in Table 1.

Most weight-loss interventions (*n* = 9) lasted 6 months [40, 41, 43, 45–50], except of one which lasted 16 weeks [42], and the Stepping Stone study whose length of duration was 12 weeks [44]. All the studies reported a follow-up phase.

Studies used a combination of widely different interventions strategies, such as in-person, telephone or mailed delivered interventions implementing either in group or individual sessions, including telephone contact, text messages, web-based platform and/or email guidance via newsletters. In more detail, in-person group sessions were included in four studies [40, 42, 44, 46], individual telephone sessions were included in three studies [47, 48, 50], in-person group meetings along with telephone individual counselling were used in two studies [43, 49], either in-person or telephone individual sessions was delivered in one study [45], and mailed intervention was delivered in one study [41]. As mentioned above, interventions’ strategies included in addition telephone contact [40, 42, 44], text messages [46, 48], web-based learning platform [47], and newsletters [40, 41, 46, 48, 50].

Self-Monitoring was the most common element of behaviour modifications in every intervention and it was implemented in all studies with food records or diaries and exercise logs. Many other tools of self-monitoring were utilised as well, including pedometers or shoes chips to record the daily steps [40–45, 48, 50], weight records [40, 45–48, 50], notebooks/workbooks/worksheets/booklets with educational material [40–45, 47–50], class activities [46], web-based resources [40, 41, 47], and digital videos [40].

#### Lifestyle modification

All the studies reported that weight loss interventions were developed with two main target behaviours: dietary and physical activity modifications.

Concerning the dietary modifications, the studies targeted firstly on an energy intake reduction and secondly on the diet quality and a healthier eating behaviour.

Upon these, seven to eleven studies reported a 500–1000 kcal daily energy deficit [40, 42, 43, 45, 46, 48, 50]. In two studies [47, 49] the energy intake depended between 1200 and 2200 kcal/day. Specifically, in Power-remote trial [47] the energy intake was 1200–2200 kcal/day depending on the initial body weight, and in Wiser trial [49] the daily caloric intake was restricted to 1200–1500 kcal/day. Finally, in two studies [41, 44] a reduced energy intake was reported without specifying the energy restriction. Extensively, in the Dames trial [41] dietary modification was either directed to lower-calorie substitutes or provided with guidance on portion control, but in the Stepping stone study [44] it was only reported a modification of diet to promote approximately 1 lb of weight loss per week, without any further details mentioned.

Healthier eating modifications varied between the eleven studies. Nine studies recommended increased daily intake of fruits and vegetables [40, 42–44, 46–50], and in six studies the recommendation was specified in at least 5 or more servings of fruits and vegetables per day [43, 44, 46–49]. Similarly, six studies recommended an increment in daily fibre or grains intake [40, 42, 43, 45, 49, 50]. Seven of the studies targeted reduction in fat intake with a dietary fat goal of at least < 35% of total energy intake [43–48, 50]. Just one study recommended a decrease in sugar consumption [45].

Physical activity modifications were implemented through various guidelines. All studies recommended an increase in aerobic exercise and four of them targeted on a daily goal of 10,000 steps [40, 44, 45, 48]. Seven of the studies additionally included strength training [40–42, 46, 48–50], of which five of them specified a muscle strengthening goal of at least 2 times per week [40–42, 48, 49]. Four studies suggested an increase in daily lifestyle physical activity [40, 42, 43, 48]. A progressive exercise intervention with a step-wise increase in time and intensity of physical activity was recommended by eight of the studies [40, 42, 45–50].

Each study determined its aerobic exercise goal differently; thus, in six studies, the aerobic exercise goal was approximately 150–200 min per week [41, 44–46, 49, 50], whereas in five studies, it was > 200 min minutes per week [40, 42, 43, 47, 48].

It is worth mentioning that 82% of the studies (*n* = 9) reported that dietary and/or exercise recommendations were in agreement with the official guidelines of several international organisations [40, 41, 43–48, 50].

#### Body weight change goal

Nine of the eleven studies reported a specific weight loss goal ranging between 5–10% of the initial body weight [40, 43–50], while in the remaining two studies the outcome goal was targeted at any body weight reduction [41, 42]. The final outcomes for the weight loss of each study are shown in Table 3.

**Table 3:** Summary of BCTs, risk of bias, weight loss outcomes and effectiveness of each study

Studies	ROB-2	Weight loss results	Promise	BCTs
Rock [40]	Some concerns	6% (12 months)	“quite promising”	19
Demark-Wahnefried [41]	Some concerns	4.6% (12 months)	“quite promising”	10
Mefferd [42]	High	6.8% (16 weeks)	“very promising”	18
Djuric [43]	High	60% of the women in the intervention reached 10% of WL. (-9.3 kg)	“very promising”	12
Sheppard [44]	High	0.8% (12 weeks)	“non-promising”	14
Harrigan [45]	Some concerns	6.4% (6 months, in-person) 5.4% (6 months, telephone)	“very promising”	11
Stolley [46]	Some concerns	3.6% (6 months)	“very promising”	17
Santa-Maria [47]	Some concerns	51% of the women in the intervention reached 5% of WL	“quite promising”	20
Reeves [48]	Some concerns	5.7% (6 months)	“very promising”	23
Schmitz [49]	Some concerns	8.6% (6 months)	“very promising”	21
Goodwin [50]	Some concerns	5.3% (6 months)	“very promising”	20

### Risk of bias

**Fig. 2:**
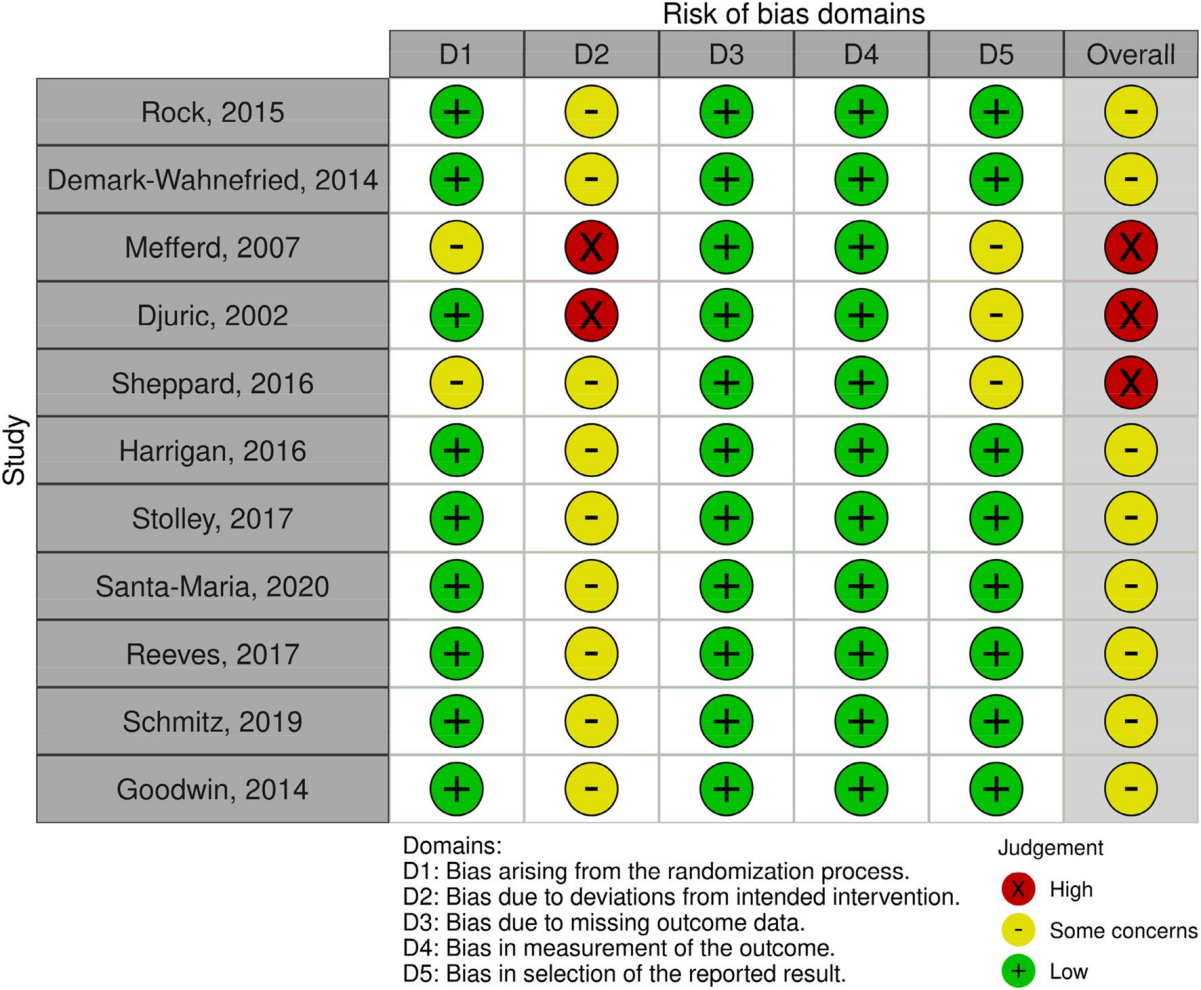
Risk of bias assessment

From the ten included studies, none had low risk of bias, three of them found at high risk and eight studies arose some concerns considering the bias. Two domains were judged to have low risk of bias in all studies, namely bias due to missing outcome data and bias in measurement of the outcome. Bias arising from the randomization process was found to be low in nine to eleven studies, while bias in selection of the reported result was found to be low in eight to eleven studies. Bias due to deviations from intended interventions was judged high risk in two studies and of some concerns in the remaining studies. Lack of blinding of participants and assessors to the interventions and non-appropriate analysis used to estimate the effect of assignment to the intervention were the most common sources of potential bias across the studies. Risk of bias judgement for each study is included in Fig. 2 [38] and in detail in supplemental file 3. Excep for the included papers, any protocol and any previous or subsequent publication of each study were considered for the extraction of results. Inter-rater reliability was substantial (61.3%; κ = 0.694, *p* < 0.001).

### Results of synthesis

#### Intervention promise

Seven studies were classified as “very promising” [42, 43, 45, 46, 48–50], three studies as “quite promising” [40, 41, 47], and only one study as “non-promising” [44]. Inter-rater agreement was moderate (81.8%; κ = 0.593, *p* < 0.004).

The “very promising” category included five of the eight studies with some concerns in the risk of bias assessment and two of the three studies with high bias, as shown in Table 3. The “quite promising” studies included three trials with some concerns about bias, while the “non-promising” study had a high bias. The heterogeneity of the studies’ characteristics discourages effective comparisons between the different categories of interventions’ promise.

#### Behaviour change theory

The most common theoretical framework used was Bandura’s Social Cognitive Theory (SCT) in ten studies, either alone [43, 45, 47, 48, 50] or in combination with other theoretical models [40, 41, 44, 46, 49]. In the ENERGY trial [40], SCT was combined with the Cognitive Behavioural Treatment of Obesity (CBT-OB), which includes cognitive therapy techniques with the goal of optimising maintenance of weight loss. In Dames trial [41], SCT was combined with the Transtheoretical Model of Change (TTM), while in the Stepping Stone study [44] with the Theory of Planned Behaviour (TPB). Finally, the Socio-Ecological Model (SEM) was additionally used in the Moving Forward trial [46], and the Behavioural Self-Management Theory in the Wiser trial [49]. Seven of the aforementioned studies [40, 44, 46–50] utilised strategies of behavioural weight loss programs, such as the Motivational Interviewing Technique (MIT).

Only one study, the Healthy Weight Management (HWM) study [42] was guided by the Cognitive Behavioural Therapy (CBT) and its components.

According to the coding of the Theory Coding Scheme, all trials mentioned the targeted theoretical constructs relevant to behaviour (item 2) and their correlation with the intervention techniques (items 7–9, 11), contrariwise no trials selected participants based on theory-related constructs (item 4). Additionally, 10 trials [40–49] reported using theory to select or develop the intervention (item 5) and 7 trials [40–45, 50] to tailor the intervention to the needs of the participants (item 6). Finally, 9 trials [40–48] linked all theory-relevant constructs to at least one intervention technique (item 10).

#### Behaviour change techniques (BCTs)

The studies varied widely in the techniques used to implement their interventions, which complicated the mapping of BCTs. Forty-two different BCTs were identified across the eleven studies. Based on the BCT taxonomy classification studies included in this review used 10–23 BCTs. The “very promising” interventions included 11–23 BCTs, the “quite promising” between 10 and 20 BCTs and the “non-promising” intervention included 14 BCTs. As shown in Table 4, the “promising” (very and quite) studies used mainly the following BCTs: In ten studies, 1.1 Goal setting (behaviour), 2.3 Self-monitoring of behaviour, 4.1 Instruction on how to perform the behaviour, and 9.1 Credible source; in nine studies, 1.2 Problem solving, and 2.2 Feedback on behaviour; in eight studies, 1.3 Goal setting (outcome), and 8.7 Graded tasks; in seven studies, 1.4 Action planning, and 6.1 Demonstration of the behaviour, and in six studies, 2.4 Self-monitoring of outcome(s) of behaviour.

**Table 4:** BCTs promise ratio

BCT’s	Time used	Promising interventions	Non promising intervention	Promise Ratio
1.1 Goal setting (behaviour)	11	10	1	10
2.3 Self-monitioring of behaviour	11	10	1	10
4.1 Instruction on how to perform the behaviour	11	10	1	10
9.1 Credible source	11	10	1	10
1.2 Problem solving	10	9	1	9
2.2 Feedback on behaviour	9	9	0	9
1.3 Goal setting (outcome)	9	8	1	8
8.7 Graded tasks	9	8	1	8
1.4 Action planning	8	7	1	7
6.1 Demonstration of the behaviour	8	7	1	7
2.4 Self-monitoring of outcome(s) of behaviour	6	6	0	6
3.3 Social support (emotional)	6	5	1	5
1.5 Review behaviour goal(s)	5	5	0	5
8.1 Behavioural practice/rehearsal	5	5	0	5
8.2 Behaviour substitution	5	5	0	5
8.4 Habit reversal	5	5	0	5
11.2 Reduce negative emotions	5	4	1	4
2.7 Feedback on outcome(s) of behaviour	4	4	0	4
3.1 Social support (unspecified)	4	4	0	4
7.1 Prompts/cues	4	4	0	4
12.3 Avoidance/Reducing exposure to cues for the behaviour	4	4	0	4
1.7 Review outcome goal(s)	3	3	0	3
5.1 Information about health consequences	3	3	0	3
8.6 Generalisation of target behaviour	3	3	0	3
13.2 Framing/reframing	3	3	0	3
8.3 Habit formation	2	2	0	2
9.2 Pros and cons	2	2	0	2
12.2 Restructuring the social environment	2	2	0	2
15.4 Self-talk	2	2	0	2

Details of the BCTs used in each study are included in supplemental file 2. Inter-rater agreement was almost perfect (98%; κ = 0.929, *p* < 0.001).

## Discussion

In this systematic review, the elements of the included interventions, such as intervention strategy (duration, sessions, tools), behaviour change models and techniques, behavioural components (diet and exercise goals) and primary outcomes (weight loss goal) were recorded. Studies differed significantly in terms of sample size, design, and interventions, but the dietary, physical activity, and weight loss goals were consistent across all trials. Interventions with larger sample sizes were assumed to be less biased and more promising. According to the findings of the present study, the “promising” interventions [40–43, 45–50] were more likely to include all or most of all of the aforementioned characteristics. Specifically, it was observed that the “promising” behavioural interventions treating obesity in breast cancer survivors had a six-month duration of treatment, followed by in-person group or individual sessions, either individual telephone sessions, or their combination. The weight loss target was at least 5% of the initial body weight, through a 500–1000 kcal daily energy deficit, tailored according to patients’ personalized energy needs. Behavioural modification aimed to increase consumption of fruits, vegetables and fibre and to reduce dietary fat along with a gradually increased exercise goal of at least 30 min per day. Self-monitoring was identified as the most important mediator of behavioural modification with the application of food record, exercise logs and pedometers as the best self-monitoring tools, and complementary tools such as weight records and educational materials. Bandura’s Social Cognitive Theory (SCT) was the theoretical framework that was most commonly used in the effective interventions, while the ten most frequently included behaviour change techniques were behaviour goal setting, self-monitoring of behaviour, instructions on how to perform the behaviour, credible source, problem solving, behaviour feedback, outcome goal setting, graded tasks, action planning, and demonstration of the behaviour.

The findings of the present systematic review are in line with the Weight Loss and Health Goals and Intervention Strategies Guidelines for the management of overweight and obesity in adults [59]. The Expert Panel of the American College of Cardiology, the American Heart Association Task Force on practice guidelines and the Obesity Society recommend that the most effective behavioural weight loss treatment is an in-person, high-intensity (≥ 14 sessions in 6 months) comprehensive weight loss intervention provided in individual or group sessions by a trained healthcare professional or a nutrition professional, with the following principal components: a moderate reduced-calorie diet designed to induce an energy deficit of ≥ 500 kcal/d, a program of increased physical activity equal to ≥ 30 min/d the most days of the week and, the use of behavioural therapy, and methods such as self-monitoring to facilitate adherence to diet and exercise guidelines [59]. These recommendations suggest that there is evidence to support that comprehensive lifestyle interventions consisting of diet, physical activity, and behavioural therapy result in optimal weight loss in 6 months with frequent, initially weekly sessions in overweight and obese individuals. Furthermore, long-term interventions or a follow-up phase of more than 2 years duration could optimize weight loss maintenance and any potentially benefits on cancer end points [60]. Concerning the mode of interventions’ delivery, the on-site (face-to-face) treatment in group or individual sessions could benefit overweight and obese individuals [59], while telephone-based or smart phone applications interventions that target lifestyle behaviour were found to be effective in cancer survivors [61].

The majority of the studies included in this review had a weight loss target of 5–10% of the initial body weight and are consistent with the findings of other reviews [10, 62]. It has been described that although sustained weight loss of as little as 3–5% of body weight may lead to clinically meaningful reductions in glycemic measures, in blood pressure and in some cardiovascular risk factors, a greater weight loss produces better health benefits [59, 63]. Moreover, the guidelines for the management of overweight and obesity in adults suggest a weight loss goal of 5–10% of baseline body weight within 6 months and continued intervention contact and support after initial weight loss treatment is associated with better maintenance of lost weight [59]. Lifestyle interventions for cancer survivors appear to still focus on outcomes related to diet, fitness and cancer-related psychosocial factors [12]. To determine the behaviours and outcome goals, most of the studies use the recommendations for lifestyle changes, consistent with dietary and physical activity guidelines for cancer survivors [64–66]. According to these guidelines, adult survivors should aim to exercise at least 150 min per week of moderate to vigorous physical activity above their usual activities, including strength training at least 2 days per week, and adapt a healthy plant-based diet with high consumption of fruits, vegetables, and whole grains to achieve an increased intake of fibre along with limited consumption of processed and red meat. A recent meta-analysis of observational studies in European countries found moderate certainty that adherence to breast cancer guidelines was associated with increased overall and disease-free survival, enhancing the rigorous adoption and implementation of breast cancer guidelines in the clinical setting [67].

Interventions underpinned by behaviour change theories and utilizing various behaviour change techniques were found to be more effective than those not based on any theory. Hence, the use of behaviour change theories and strategies is highly recommended by many official guidelines [3]. The American Dietetic Association encourage health care professionals to practice behavioural therapy for planning effective nutrition counselling interventions [14]. Although, the use of a behavioural therapy is suggested, it remains unclear which theory is the most effective improving the participants’ behaviour, according to a recent review article about the use of behaviour change theories in lifestyle interventions for cancer survivors [22]. In the present review, SCT was the most frequently used theory, similarly to the findings of other reviews of behaviour change interventions targeting obesity in cancer survivors [22, 24, 62]. Michie et al. suggested that using a theory to influence intervention effectiveness should be combined with the appropriate intervention components [30]. Behaviour Change Techniques (BCTs) used in RCTs are auxiliary for the identification of the strategies implemented in each intervention. It has been found that goal setting (1.3), problem solving (1.2) and social support (3.1) along with self-monitoring (2.3) are effective behavioural change techniques to lose weight in a helpful and manageable manner [13, 14]. Additionally with these four BCTs, credible source (9.1), instruction on how to perform the behaviour (4.1) and feedback of behaviour (2.2) were the most commonly used strategies in weight loss interventions for cancer survivors [22, 24, 62]. The importance of the role of a credible source, such as oncology counselor, has been highlighted in providing evidence-based information on the association of breast cancer and diet and physical activity in daily life in developing interventions effectively [68], and additionally in identifying the most important determinants of lifestyle changes in cancer survivors [69]. Moreover, it has been suggested that weight management interventions delivered by healthcare professionals can be effective for weight loss for up to 6 months [70]. The current findings concerning BCTs are in agreement with the aforementioned outcomes.

The findings of the present systematic review should be considered under the light of its strengths and limitations. Regarding the strengths, two independent reviewers performed the selection and the rating process and a third independent reviewer solved any disagreements. Secondly, the PRISMA guidelines for reporting a systematic review were followed [27] and the methodological quality of each study was assessed based on the Cochrane risk of bias tool for randomized trials (RoB 2) [37]. Moreover, only RCT studies were included to maximize the quality of the studies being reviewed and the BCT taxonomy was used [34] to code the interventions. This measure makes the behavioural interventions comparable and allows future researchers to review methods used in detail. Regarding the limitations, the included publications were in English language, so non-English articles may have been missed. There is also a high risk of publication bias. All of the included studies were RCTs, and ten to eleven interventions (90.9%) were found “promising” compared to a solely “non-promising” intervention. “Promising” interventions are more likely to be published, which may imply that “non-promising” interventions found in non-RCT trials were not considered in this review, skewing the results. No meta-analysis was conducted in the present review due to the large heterogeneity of the included studies, thus quantitative approaches, such as regression-based assessments for determining whether there is a skew to effect, were not assessed, nor were the moderator effects of publication bias, as is recommended. Since such tests generally assume a single population size effect, inferences of publication bias are dangerous in the face of heterogeneity [71]. Additionally, coding of the theory coding scheme and the behaviour change techniques depended on the reporting quality, quantity and accuracy within the RCTs, and these varied considerably. Although the current review was based on the publications of each study, the majority of the studies referred to a protocol paper and the Authors tried to contact the corresponding authors of the trials that did not have a full description of the intervention [42, 43]. Only one author was reached [43], therefore any details concerning the interventions’ content may be lacking. Finally, in this review is that most studies were rated as having some concerns or a high risk of bias due to the lack of blinding of participants and assessors, suggesting that the body of evidence presented should be carefully considered.

## Conclusions

Considering the unique needs of breast cancer survivors, lifestyle interventions should include behavior modifications in diet, physical activity and psychosocial factors according to the official guidelines with the use of behavioural theory and the suitable behavioural change techniques. The completion of the initial cancer treatment signals a critical period, in which breast cancer survivors may adopt healthy behaviours thus maintain or adopt a healthy body weight. The identification of the optimal methods targeting obesity seems vital for lowering the risk of cancer recurrence and overall mortality. The taxonomy can help with the understanding of behaviour change tools used in the interventions; thus, the need for stronger identification of the components of the intervention using a taxonomy of behavioural change techniques should be highlighted. The findings of this systematic review may enable healthcare professionals to include the reported elements and BCTs in their behavioural interventions in order to help breast cancer survivors to improve their lifestyle, control their body weight and maintain the changes more effectively.

## Supplementary Information

Below is the link to the electronic supplementary material.

Supplementary file1 (PDF 201 KB)

Supplementary file2 (PDF 435 KB)

Supplementary file3 (PDF 162 KB)

## Data Availability

All datasets generated during and/or analysed during the current study are available from the corresponding author on request.
