# Supplementary material for: Identifying the effective behaviour change techniques in nutrition and physical activity interventions for the treatment of overweight/obesity in post-treatment breast cancer survivors: a systematic review
Source: Cancer Causes Control. 2023 May 6;34(8):683–703. doi: 10.1007/s10552-023-01707-w (PMC10267275; doi:10.1007/s10552-023-01707-w)
Supplement: Supplementary file 1 — Supplementary file1 (PDF 201 KB) [file 10552_2023_1707_MOESM1_ESM.pdf]

Database Search Strategies

Pubmed/Medline

#1	breast neoplasm[MeSH Terms]
#2	(breast cancer) OR (breast cancer survivors)
#3	(#1) OR (#2)
#4	((((lifestyle intervention) OR (behavioral intervention)) OR (behavioural intervention)) OR (theory-based intervention)) OR (theoretical framework)
#5	(#3) AND (#4)
#6	(overweight) OR (obesity)
#7	(#5) AND (#6)
#8	((((weight loss) OR (weight-loss)) OR (weight change)) OR (weight management)) OR (BMI reduction)
#9	(#7) AND (#8)
Results	281
Last search	01/08/2022

Central/Cochrane Library

#1	MeSH descriptor: [Breast Neoplasms] explode all trees
#2	(breast cancer): ti,ab,kw
#3	breast cancer survivors
#4	#1 or #2 or #3
#5	(lifestyle intervention): ti,ab,kw
#6	(behavioral intervention): ti,ab,kw
#7	(behavioural intervention): ti,ab,kw
#8	(theory-based intervention): ti,ab,kw
#9	(theoretical framework): ti,ab,kw
#10	#5 or #6 or #7 or #8 or #9
#11	#4 and #10
#12	(overweight): ti,ab,kw
#13	(obesity): ti,ab,kw
#14	#12 and #13
#15	#11 and #14
#16	(weight loss): ti,ab,kw

#17	(weight-loss): ti,ab,kw
#18	(weight change): ti,ab,kw
#19	(weight management): ti,ab,kw
#20	(BMI reduction): ti,ab,kw
#21	#16 or #17 or #18 or #19 or #20
#22	#15 and #21
Results	142
Last search	01/08/2022

Scopus Database

(TITLE-ABS-KEY (breast AND cancer) OR TITLE-ABS-KEY (breast AND cancer AND survivors) AND TITLE-ABS-KEY (lifestyle AND intervention) OR TITLE-ABS-KEY (behavioral AND intervention) OR TITLE-ABS-KEY (behavioural AND intervention) OR TITLE-ABS-KEY (theory-based AND intervention) OR TITLE-ABS-KEY (theoretical AND framework) AND TITLE-ABS-KEY (overweight) OR TITLE-ABS-KEY (obesity) AND TITLE-ABS-KEY (weight AND loss) OR TITLE-ABS-KEY (weight-loss) OR TITLE-ABS-KEY (weight AND change) OR TITLE-ABS-KEY (weight AND management) OR TITLE-ABS-KEY (bmi AND reduction))
Results: 220
Last search: 01/08/2022

TripDatabase

<u>Simple Search</u>
(breast cancer OR breast cancer survivors) AND (lifestyle intervention OR behavioral intervention OR behavioural intervention OR theory-based intervention OR theoretical framework) AND (overweight OR obesity) AND (weight loss OR weight-loss OR weight change OR weight management OR BMI reduction)

Last search: 01/08/2022
Total Results 1226
Systematic Reviews 89
Evidence Based Synopses 106
Guidelines 400
Regulatory Guidance 30
Key primary research 4
clinical Q&A 2
Controlled Trials 37
Primary research 124
Ongoing Clinical Trials 177
Ongoing systematic reviews 1
patients information leaflets 5
blogs 19
eTextbooks 277

62 publications excluded with a reason

**Publications**	**Reasons**
Allicock, 2021	Non theory-based
Anderson, 2016	Other results of the LEAN study

Publications	Reasons
Appel, 2011	Protocol study (POWER trial)
Arikawa, 2018	Non theory-based
Beford, 2014	Protocol study
Beford, 2016	Randomization after 6 months / wrong study design
Campbell, 2012	Non theory-based
Christifano, 2016	Non theory-based
Delahanty, 2021	Protocol study (BWEL Study)
Demark-Wahnefried, 2015	Other results of the Energy Trial (examining quality of life)
Di Meglio, 2022	Protocol study (MEDEA study design)
Dittus, 2015	Non theory-based
Dittus, 2018	Non theory-based
Djuric, 2010	Randomization after 6 months / wrong study design
Fazzino, 2016	The aim of the intervention was not weight loss
Ferrucci, 2017	Other results of the LEAN study
Gao, 2019	The aim of the intervention was not weight loss
Ghavami, 2015	Non theory-based
Gnagnarella, 2016	Protocol study (In Forma Study)
Goodwin, 2019	Other results of the LISA trial
Goodwin, 2020	Other results of the LISA trial
Greenlee, 2013	Non theory-based
Greenlee, 2022	Not overweight/obese
Harris, 2013	Non RCT
Hauner, 2020	Protocol study (SUCCESS C Study)
Hooshmand, 2021	Non theory-based
Janni, 2019	Initial results / not the intervention results (SUCCESS C Study)
Jen, 2004	Other results of the trial of Djuruc et al, 2002
Kim, 2011	Not overweight/obese
Kwarteng, 2022	Other results of the Moving Forward study
Lisevick, 2021	Other results of the LEAN study
Long Parma, 2022	The aim of the intervention was not weight loss

Publications	Reasons
Lozano-Lozano, 2020	Non theory-based
Lyons, 2016	Non theory-based
Male, 2022	Single arm study / not control group
Nguyen, 2021	Other results of the LEAN study
Parekh, 2018	Not overweight/obese
Patterson, 2016	Pharmacological RCT (Reach for Health Trial)
Puklin, 2021	Other results of the LEAN study
Quintiliani, 2016	Single arm study / not control group
Ramirez, 2017	Protocol study
Ream, 2022	The aim of the intervention was not weight loss
Reeves, 2012	Other results of the Living Well Trial
Reeves, 2016	Study Design/protocol study
Rock, 2013	Study Design/protocol study
Sanft, 2014	Other results of the LEAN study
Sanft, 2016	Other results of the LEAN study
Sanft, 2018	Other results of the LEAN study
Saxton, 2014	Non theory-based
Scott, 2013	Non theory-based
Sedgo, 2016	Other results of the Energy Trial (examining comorbidities)
Sheng, 2022	Other results of the POWER-remote trial
Stolley, 2009	Other results of the Moving Forward study (not an intervention)
Stolley, 2015	Study Design/protocol study
Sturgeon, 2017	Other results of the Wiser Trial
Sturgeon, 2018	Other results of the Wiser Trial
Sweeney, 2020	Non theory-based
Travier, 2014	Non theory-based
Vitolins, 2014	Single arm study / not control group
Vona-Devis, 2015	Non theory-based
Winkels, 2017	Describes the protocol
Zuniga, 2019	The aim of the intervention was not weight loss

References of the excluded studies

1. Allicock, M., Kendzor, D., Sedory, A., Gabriel, K. P., Swartz, M. D., Thomas, P., Yudkin, J. S., & Rivers, A. (2021). A Pilot and Feasibility Mobile Health Intervention to Support Healthy Behaviors in African American Breast Cancer Survivors. J Racial Ethn Health Disparities, 8(1), 157-165. https://doi.org/10.1007/s40615-020-00767-x
2. Anderson, C., Harrigan, M., George, S. M., Ferrucci, L. M., Sanft, T., Irwin, M. L., & Cartmel, B. (2016). Changes in diet quality in a randomized weight loss trial in breast cancer survivors: The lifestyle, exercise, and nutrition (LEAN) study [Article]. npj Breast Cancer, 2(1), Article 16026. https://doi.org/10.1038/npjbcancer.2016.26.
3. Appel L, Clark J, Yeh H, Wang N, Coughlin J, Daumit G, et al. Comparative effectiveness of weight-loss interventions in clinical practice. N Engl J Med 2011; 365:1959–68. https://doi.org/10.1056/nejmoa1108660.
4. Arikawa, A. Y., Kaufman, B. C., Raatz, S. K., & Kurzer, M. S. (2018). Effects of a parallel-arm randomized controlled weight loss pilot study on biological and psychosocial parameters of overweight and obese breast cancer survivors. Pilot Feasibility Stud, 4, 17. https://doi.org/10.1186/s40814-017-0160-9
5. Befort, C., Klemp, J., Fabian, C., Perri, M., Sullivan, D., Schmitz, K., Diaz, F., & Shireman, T. (2014). Protocol and recruitment results from a randomized controlled trial comparing group phone-based versus newsletter interventions for weight loss maintenance among rural breast cancer survivors [Article]. Contemporary Clinical Trials, 37(2), 261-271. https://doi.org/10.1016/j.cct.2014.01.010
6. Befort, C. A., Klemp, J. R., Sullivan, D. K., Shireman, T., Diaz, F. J., Schmitz, K., Perri, M. G., & Fabian, C. (2016). Weight loss maintenance strategies among rural breast cancer survivors: the rural women connecting for better health trial [Journal Article; Randomized Controlled Trial]. Obesity (Silver Spring, Md.), 24(10), 2070-2077. https://doi.org/10.1002/oby.21625
7. Campbell, K. L., Van Patten, C. L., Neil, S. E., Kirkham, A. A., Gotay, C. C., Gelmon, K. A., & McKenzie, D. C. (2012). Feasibility of a lifestyle intervention on body weight and serum biomarkers in breast cancer survivors with overweight and obesity. J Acad Nutr Diet, 112(4), 559-567. https://doi.org/10.1016/j.jada.2011.10.022
8. Christifano, D. N., Fazzino, T. L., Sullivan, D. K., & Befort, C. A. (2016). Diet Quality of Breast Cancer Survivors after a Six-Month Weight Management Intervention: Improvements and Association with Weight Loss [Article]. Nutrition and Cancer, 68(8), 1301-1308. https://doi.org/10.1080/01635581.2016.1224368
9. Delahanty, L. M., Wadden, T. A., Goodwin, P. J., Alfano, C. M., Thomson, C. A., Irwin, M. L., Neuhouser, M. L., Crane, T. E., Frank, E., Spears, P. A., & et al. (2022). The Breast Cancer Weight Loss trial (Alliance A011401): a description and evidence for the lifestyle intervention [Journal: Review]. Obesity (Silver Spring, Md.), 30(1), 28-38. https://doi.org/10.1002/oby.23287
10. Demark-Wahnefried, W., Colditz, G. A., Rock, C. L., Sedjo, R. L., Liu, J., Wolin, K. Y., Krontiras, H., Byers, T., Pakiz, B., Parker, B. A., Naughton, M., Elias, A., & Ganz, P. A. (2015). Quality of life outcomes from the Exercise

and Nutrition Enhance Recovery and Good Health for You (ENERGY)-randomized weight loss trial among breast cancer survivors. Breast Cancer Res Treat, 154(2), 329-337. https://doi.org/10.1007/s10549-015-3627-5

11. Di Meglio, A., Martin, E., Crane, T. E., Charles, C., Barbier, A., Raynard, B., Mangin, A., Tredan, O., Bouleuc, C., Cottu, P. H., & et al. (2022). A phase III randomized trial of weight loss to reduce cancer-related fatigue among overweight and obese breast cancer patients: MEDEA Study design [Clinical Trial, Phase III; Journal Article; Multicenter Study; Randomized Controlled Trial]. Trials, 23(1), 193. https://doi.org/10.1186/s13063-022-06090-6
12. Dittus, L.K. (2015). Effectiveness of an Internet Delivered Behavioral Weight Loss Intervention Provided to Breast Cancer Survivors. Advances in Obesity, Weight Management & Control. 2(3).
13. Dittus, K. L., Harvey, J. R., Bunn, J. Y., Kokinda, N. D., Wilson, K. M., Priest, J., & Pratley, R. E. (2018). Impact of a behaviorally-based weight loss intervention on parameters of insulin resistance in breast cancer survivors. BMC Cancer, 18(1), 351. https://doi.org/10.1186/s12885-018-4272-2
14. Djuric, Z., Ellsworth, J., Rapai, M., Weldon, A., Kim, J., & Sen, A. (2010). A diet and exercise intervention in women being treated for breast cancer. Cancer research. https://www.cochranelibrary.com/central/doi/10.1002/central/CN-01984892/full
15. Fazzino, T. L., Fleming, K., & Befort, C. (2016). Alcohol Intake Among Breast Cancer Survivors: Change in Alcohol Use During a Weight Management Intervention. JMIR Cancer, 2(2), e15. https://doi.org/10.2196/cancer.6295
16. Ferrucci, L. M., Cartmel, B., Harrigan, M., Sanft, T., Playdon, M., Jia, W., Yu, H., Johnson, C. H., Pusztai, L., Chagpar, A. B., & et al. (2017). Metabolomics and body mass index among breast cancer survivors in The Lifestyle, Exercise, and Nutrition (LEAN) Study [Journal: Conference Abstract]. Cancer research, 77(13). https://doi.org/10.1158/1538-7445.AM2017-5321
17. Gao, H., Cohen, E. N., Yang, P., Austin, T. A., Haddad, R., Wu, Q., Basen-Engquist, K. M., Ochoa, J. M., Arun, B. K., Perkins, G. H., & et al. (2019). Circulating tumor cell subset analysis to assess lifestyle interventions for breast cancer patients after neoadjuvant chemotherapy [Journal: Conference Abstract]. Cancer research, 79(4). https://doi.org/10.1158/1538-7445.SABCS18-P3-01-15
18. Ghavami, H., & Akyolcu, N. (2015). Effects of lifestyle interventions on body mass index in breast cancer patients [Journal: Conference Abstract]. Value in health., 18(7), A433. https://www.cochranelibrary.com/central/doi/10.1002/central/CN-01164136/full
19. Gnagnarella, P., Dragà, D., Baggi, F., Simoncini, M. C., Sabbatini, A., Mazzocco, K., Bassi, F. D., Pravettoni, G., & Maisonneuve, P. (2016). Promoting weight loss through diet and exercise in overweight or obese breast cancer survivors (InForma): study protocol for a randomized controlled trial. Trials, 17, 363. https://doi.org/10.1186/s13063-016-1487-x
20. Goodwin, P. J., Segal, R., Vallis, M., Ligibel, J. A., Pond, G. R., Robidoux, A., Findlay, B. P., Gralow, J. R., Mukherjee, S. D., Levine, M. N., & et al. (2019). Lifestyle intervention study (LISA) in early breast cancer (BC):

an RCT of the effects of a telephone-based weight loss intervention (with educational materials) vs educational materials alone on disease-free survival (DFS) [Journal: Conference Abstract]. Cancer research, 79(4). https://doi.org/10.1158/15387445.SABCS18-PD6-04

21. Goodwin, P. J., Segal, R. J., Vallis, M., Ligibel, J. A., Pond, G. R., Robidoux, A., Findlay, B., Gralow, J. R., Mukherjee, S. D., Levine, M., & et al. (2020). The LISA randomized trial of a weight loss intervention in postmenopausal breast cancer [Journal: Article]. npj Breast Cancer, 6(1). https://doi.org/10.1038/s41523-020-0149-z
22. Greenlee, H.A., Crew, K.D., Mata, J.M., McKinley, P.S., Rundle, A.G., Zhang, W., Liao, Y., Tsai, W.Y., Hershman, D.L. (2013). A pilot randomized controlled trial of a commercial diet and exercise weight loss program in minority breast cancer survivors. Obesity (Silver Spring), 21(1):65-76. doi: 10.1002/oby.20245. PMID: 23505170; PMCID: PMC4705911.
23. Greenlee, H., Santiago-Torres, M., Koch, P., Tsai, W. Y., Gray, H. L., Brickman, A. M., Gaffney, A. O., Eddy, M., Thomson, C. A., Crane, T. E., & et al. (2022). A randomized, controlled, 2x2 factorial tria of a diet and physical activity intervention among Latina breast cancer survivors: ¡mi Vida Saludable! study [Journal: Conference Abstract]. Cancer research, 82(4 SUPPL). https://doi.org/10.1158/1538-7445.SABCS21-P1-10-07
24. Harris, M., Swift, D., Myers, V., Earnest, C., Johannsen, N., Champagne, C., Parker, B., Levy, E., Cash, K., & Church, T. (2013). Cancer Survival Through Lifestyle Change (CASTLE): a Pilot Study of Weight Loss [Article]. International Journal of Behavioral Medicine, 20(3), 403-412. https://doi.org/10.1007/s12529-012-9234-5
25. Hauner, D., Rack, B., Friedl, T., Hepp, P., Janni, W, Hauner, H. (2020). Rationale and description of a lifestyle intervention programme to achieve moderate weight loss in women with non-metastatic breast cancer: the lifestyle intervention part of the SUCCESS C Study. BMJ Nutr Prev Health, 3(2):213-219. doi: 10.1136/bmjnph-2020-000119. PMID: 33521531; PMCID: PMC7841841.
26. Hooshmand Moghadam, B., Golestani, F., Bagheri, R., Cheraghloo, N., Eskandari, M., Wong, A., Nordvall, M., Suzuki, K., Pournemati, P. (2021). The Effects of High-Intensity Interval Training vs. Moderate-Intensity Continuous Training on Inflammatory Markers, Body Composition, and Physical Fitness in Overweight/Obese Survivors of Breast Cancer: A Randomized Controlled Clinical Trial. *Cancers*, *13*, 4386. https://doi.org/10.3390/cancers13174386
27. Janni, W., Rack, B. K., Friedl, T. W., Muller, V., Lorenz, R., Rezai, M., Tesch, H., Heinrich, G., Andergassen, U., Harbeck, N., & et al. (2019). Lifestyle intervention and effect on disease-free survival in early breast cancer Pts: interim analysis from the randomized SUCCESS C study [Journal: Conference Abstract]. Cancer research, 79(4). https://doi.org/10.1158/15387445.SABCS18-GS5-03
28. Jen, K.L., Djuric, Z., DiLaura, N.M., Buison, A., Redd, J.N., Maranci, V., Hryniuk, W.M. (2004). Improvement of metabolism among obese breast cancer survivors in differing weight loss regimens. Obes Res, 12(2):306-12. doi: 10.1038/oby.2004.38. PMID: 14981223.

29. Kim, S.H., Shin, M.S., Lee, H.S., Lee, E.S., Ro, J.S., Kang, H.S., Kim, S.W., Lee, W.H., Kim, H.S., Kim, C.J., Kim, J., Yun, Y.H. (2011). Randomized pilot test of a simultaneous stage-matched exercise and diet intervention for breast cancer survivors. Oncol Nurs Forum, 38(2):E97-106. doi: 10.1188/11.ONF.E97-E106. PMID: 21356647.
30. Kwarteng, J. L., Matthews, L., Banerjee, A., Sharp, L. K., Gerber, B. S., & Stolley, M. R. (2022). The association of stressful life events on weight loss efforts among African American breast cancer survivors. J Cancer Surviv, 16(3), 604-613. https://doi.org/10.1007/s11764-021-01054-2
31. Lisevick, A., Cartmel, B., Harrigan, M., Li, F., Sanft, T., Fogarasi, M., Irwin, M. L., & Ferrucci, L. M. (2021). Effect of the Lifestyle, Exercise, and Nutrition (LEAN) Study on Long-Term Weight Loss Maintenance in Women with Breast Cancer [Journal Article; Randomized Controlled Trial]. Nutrients, 13(9). https://doi.org/10.3390/nu13093265
32. Long Parma, D. A., Reynolds, G. L., Muñoz, E., & Ramirez, A. G. (2022). Effect of an anti-inflammatory dietary intervention on quality of life among breast cancer survivors. Support Care Cancer, 30(7), 5903-5910. https://doi.org/10.1007/s00520-022-07023-4
33. Lozano-Lozano, M., Martín-Martín, L., Galiano-Castillo, N., Fernández-Lao, C., Cantarero-Villanueva, I., López-Barajas, I. B., & Arroyo-Morales, M. (2020). Mobile health and supervised rehabilitation versus mobile health alone in breast cancer survivors: randomized controlled trial [Journal Article; Randomized Controlled Trial]. Annals of physical and rehabilitation medicine, 63(4), 316-324. https://doi.org/10.1016/j.rehab.2019.07.007
34. Lyons, E. J., Baranowski, T., Basen-Engquist, K. M., Lewis, Z. H., Swartz, M. C., Jennings, K., & Volpi, E. (2016). Testing the effects of narrative and play on physical activity among breast cancer survivors using mobile apps: study protocol for a randomized controlled trial [Journal Article; Randomized Controlled Trial; Research Support, N.I.H., Extramural; Research Support, Non-U.S. Gov't; Research Support, U.S. Gov't, Non-P.H.S.; Research Support, U.S. Gov't, P.H.S.]. BMC Cancer, 16, 202. https://doi.org/10.1186/s12885-016-2244-y
35. Male, D., Fergus, K., & Yufe, S. (2022). 'Weighing' Losses and Gains: Evaluation of the Healthy Lifestyle Modification After Breast Cancer Pilot Program [Article]. Frontiers in Psychology, 13, Article 814671. https://doi.org/10.3389/fpsyg.2022.814671
36. Nguyen, T., Irwin, M. L., Dewan, A. T., Cartmel, B., Harrigan, M., Ferrucci, L. M., Sanft, T., Li, F., Lu, L., & Salinas, Y. D. (2021). Examining the effect of obesity-associated gene variants on breast cancer survivors in a randomized weight loss intervention [Journal Article; Randomized Controlled Trial]. Breast cancer research and treatment, 187(2), 487-497. https://doi.org/10.1007/s10549-021-06151-5
37. Parekh, N., Jiang, J., Buchan, M., Meyers, M., Gibbs, H., Krebs, P. (2018). Nutrition Literacy among Cancer Survivors: Feasibility Results from the Healthy Eating and Living Against Breast Cancer (HEAL-BCa) Study: a Pilot Randomized Controlled Trial. J Cancer Educ, 33(6):1239-1249. doi: 10.1007/s13187-017-1238-z. PMID: 28624990.

38. Patterson, R. E., Marinac, C. R., Natarajan, L., Hartman, S. J., Cadmus-Bertram, L., Flatt, S. W., Li, H., Parker, B., Oratowski-Coleman, J., Villaseñor, A., Godbole, S., & Kerr, J. (2016). Recruitment strategies, design, and participant characteristics in a trial of weight-loss and metformin in breast cancer survivors. Contemp Clin Trials, 47, 64-71. https://doi.org/10.1016/j.cct.2015.12.009
39. Puklin, L., Cartmel, B., Harrigan, M., Lu, L., Li, F. Y., Sanft, T., & Irwin, M. L. (2021). Randomized trial of weight loss on circulating ghrelin levels among breast cancer survivors. npj Breast Cancer, 7(1), 49. https://doi.org/10.1038/s41523-021-00260-6
40. Quintiliani, L. M., Mann, D. M., Puputti, M., Quinn, E., & Bowen, D. J. (2016). Pilot and Feasibility Test of a Mobile Health-Supported Behavioral Counseling Intervention for Weight Management Among Breast Cancer Survivors. JMIR Cancer, 2(1). https://doi.org/10.2196/cancer.5305
41. Ramirez, A.G., Parma, D.L., Muñoz, E., Mendoza, K.D., Harb, C., Holden, A.E.C., Wargovich, M. (2017). An anti-inflammatory dietary intervention to reduce breast cancer recurrence risk: Study design and baseline data. Contemp Clin Trials, 57:1-7. doi: 10.1016/j.cct.2017.03.009. Epub 2017 Mar 22. PMID: 28342988; PMCID: PMC6110085.
42. Ream, M., Saez-Clarke, E., Taub, C., Diaz, A., Frasca, D., Blomberg, B. B., & Antoni, M. H. (2022). Brief Post-Surgical Stress Management Reduces Pro-Inflammatory Cytokines in Overweight and Obese Breast Cancer Patients Undergoing Primary Treatment. Front Biosci (Landmark Ed), 27(5), 148. https://doi.org/10.31083/j.fbl2705148
43. Reeves, M., Winkler, E., McCarthy, N., Lawler, S., Eakin, E., & Healy, G. (2012). Living well after breast cancer: changes in objectively-measured physical activity in a weight loss trial [Journal: Conference Abstract]. Journal of science and medicine in sport, 15, S334. https://doi.org/10.1016/j.jsams.2012.11.811.
44. Reeves MM, Terranova CO, Erickson J, Job JR, Brookes DS, McCarthy N, et al. Living Well after Breast Cancer randomized controlled trial protocol: evaluating a telephone-delivered weight loss intervention versus usual care in women following treatment for breast cancer. BMC Cancer. 2016;16:830. https://doi.org/10.1186/s12885-016-2858-0.
45. Rock CL, Byers TE, Colditz GA, Demark-Wahnefried W, Ganz PA, Wolin KY, et al. Reducing breast cancer recurrence with weight loss, a vanguard trial: the Exercise and Nutrition to Enhance Recovery and Good Health for You (ENERGY) Trial. Contemp Clin Trials. 2013;34(2):282-95. https://doi.org/10.1016/j.cct.2012.12.003.
46. Sanft, T. B., Harrigan, M., Cartmel, B., Playdon, M., Zhou, Y., Loftfield, E., & Irwin, M. (2014). Effect of weight history on ability to lose weight after a 6-month randomized controlled weight loss trial in overweight breast cancer survivors: the lifestyle, exercise, and nutrition (LEAN) study [Journal: Conference Abstract]. Journal of Clinical Oncology, 32(15 SUPPL. 1). https://www.cochranelibrary.com/central/doi/10.1002/central/CN-01055970/full
47. Sanft, T., Lu, L., Harrigan, M., Cartmel, B., Zhou, Y., Chagpar, A., Pusztai, L., & Irwin, M. (2016). Randomized controlled trial of weight loss vs. usual care on telomere length in women with breast cancer: the lifestyle, exercise

and nutrition (LEAN) study [Journal: Conference Abstract]. Cancer research, 76(4). https://doi.org/10.1158/1538-7445.SABCS15-P3-08-01

48. Sanft, T., Usiskin, I., Harrigan, M., Cartmel, B., Lu, L., Li, F. Y., Zhou, Y., Chagpar, A., Ferrucci, L. M., Pusztai, L., & et al. (2018). Randomized controlled trial of weight loss versus usual care on telomere length in women with breast cancer: the lifestyle, exercise, and nutrition (LEAN) study [Journal Article; Randomized Controlled Trial]. Breast cancer research and treatment, 172(1), 105-112. https://doi.org/10.1007/s10549-018-4895-7
49. Saxton, J. M., Scott, E. J., Daley, A. J., Woodroofe, M., Mutrie, N., Crank, H., Powers, H. J., & Coleman, R. E. (2014). Effects of an exercise and hypocaloric healthy eating intervention on indices of psychological health status, hypothalamic-pituitary-adrenal axis regulation and immune function after early-stage breast cancer: a randomised controlled trial [Journal Article; Randomized Controlled Trial; Research Support, Non-U.S. Gov't]. Breast cancer research, 16(2), R39. https://doi.org/10.1186/bcr3643
50. Scott, E., Daley, A. J., Doll, H., Woodroofe, N., Coleman, R. E., Mutrie, N., Crank, H., Powers, H. J., & Saxton, J. M. (2013). Effects of an exercise and hypocaloric healthy eating program on biomarkers associated with long-term prognosis after early-stage breast cancer: a randomized controlled trial. Cancer Causes Control, 24(1), 181-191. https://doi.org/10.1007/s10552-012-0104-x
51. Sedjo, R., Flatt, S., Byers, T., Colditz, G., Demark-Wahnefried, W., Ganz, P., Wolin, K., Elias, A., Krontiras, H., Liu, J., Naughton, M., Pakiz, B., Parker, B., Wyatt, H., & Rock, C. (2016). Impact of a behavioral weight loss intervention on comorbidities in overweight and obese breast cancer survivors [Article]. Supportive Care in Cancer, 24(8), 3285-3293. https://doi.org/10.1007/s00520-016-3141-2
52. Sheng, J. Y., Santa-Maria, C. A., Blackford, A. L., Lim, D., Carpenter, A., Smith, K. L., Cohen, G. I., Coughlin, J., Appel, L. J., Stearns, V., & Snyder, C. (2022). The impact of weight loss on physical function and symptoms in overweight or obese breast cancer survivors: results from POWER-remote. J Cancer Surviv, 16(3), 542-551. https://doi.org/10.1007/s11764-021-01049-z
53. Stolley, M. R., Sharp, L. K., Oh, A., & Schiffer, L. (2009). A weight loss intervention for African American breast cancer survivors, 2006 [Article]. Preventing Chronic Disease, 6(1), Article A22. https://www.scopus.com/inward/record.uri?eid=2-s2.0-59149099227&partnerID=40&md5=386e64e905bb555cae8987825cccc6be.
54. Stolley MR, Sharp LK, Fantuzzi G, Arroyo C, Sheean P, Schiffer L, et al. Study design and protocol for moving forward: a weight loss intervention trial for African-American breast cancer survivors. BMC Cancer. 2015;15:1018. https://doi.org/10.1186/s12885-015-2004-4.
55. Sturgeon, K. M., Dean, L. T., Heroux, M., Kane, J., Bauer, T., Palmer, E., Long, J., Lynch, S., Jacobs, L., Sarwer, D. B., Leonard, M. B., & Schmitz, K. (2017). Commercially available lifestyle modification program: randomized controlled trial addressing heart and bone health in BRCA1/2+ breast cancer survivors after risk-reducing salpingo-oophorectomy. J Cancer Surviv, 11(2), 246-255. https://doi.org/10.1007/s11764-016-0582-z

56. Sturgeon, K., Foo, W., Heroux, M., & Schmitz, K. (2018). Change in Inflammatory Biomarkers and Adipose Tissue in BRCA1/2(+) Breast Cancer Survivors Following a Yearlong Lifestyle Modification Program [Article]. Cancer Prevention Research, 11(9), 545-550. https://doi.org/10.1158/1940-6207.CAPR-18-0098
57. Sweeney, F. C., Demark-Wahnefried, W., Courneya, K. S., Tripathy, D., Sami, N., Lee, K., Buchanan, T. A., Spicer, D., Bernstein, L., Mortimer, J. E., & et al. (2020). Ethnocentric differences in sarcopenicobesity and body composition in response to an aerobic and resistance exercise intervention for breast cancersurvivors [Journal: Conference Abstract]. Cancer epidemiology biomarkers and prevention, 29(6 SUPPL 1). https://doi.org/10.1158/1538-7755.DISP18-C123
58. Travier, N., Fonseca-Nunes, A., Javierre, C., Guillamo, E., Arribas, L., Peiró, I., Buckland, G., Moreno, F., Urruticoechea, A., Oviedo, G. R., Roca, A., Hurtós, L., Ortega, V., Muñoz, M., Garrigós, L., Cirauqui, B., Del Barco, S., Arcusa, A., Seguí, M. A., Borràs, J. M., Gonzalez, C. A., & Agudo, A. (2014). Effect of a diet and physical activity intervention on body weight and nutritional patterns in overweight and obese breast cancer survivors [Article]. Medical Oncology, 31(1), Article 783. https://doi.org/10.1007/s12032-013-0783-5
59. Vitolins, M. Z., Milliron, B. J., Hopkins, J. O., Fulmer, A., Lawrence, J., Melin, S., & Case, D. (2014). Weight Loss Intervention in Survivors of ER/PR-negative Breast Cancer. Clin Med Insights Womens Health, 7, 17-24. https://doi.org/10.4137/cmwh.S13955
60. Vona-Davis, L., Abraham, J., Bonner, D., Gilleland, D., Hobbs, G., Kurian, S., Yanosik, M. A., & Swisher, A. (2015). Effect of a 12-week supervised physical activity and healthy eating program on body weight, functional capacity and serum biomarkers in survivors of triple-negative breast cancer: a randomized, controlled trial [Journal: Conference Abstract]. Cancer research, 75(9). https://doi.org/10.1158/1538-7445.SABCS14-P1-09-12.
61. Winkels RM, Sturgeon KM, Kallan MJ, Dean LT, Zhang Z, Evangelisti M, et al. The Women in Steady Exercise Research (WISER) Survivor trial: the innovative transdisciplinary design of a randomized controlled trial of exercise and weight-loss interventions among breast cancer survivors with lymphedema. Contemp Clin Trials. 2017;61:63-72. https://doi.org/10.1016/j.cct.2017.07.017.
62. Zuniga, K.E., Parma, D.L., Muñoz, E., Spaniol, M., Wargovich, M., Ramirez, A.G. (2019). Dietary intervention among breast cancer survivors increased adherence to a Mediterranean-style, anti-inflammatory dietary pattern: the Rx for Better Breast Health Randomized Controlled Trial. Breast Cancer Res Treat, 173(1):145-154. doi: 10.1007/s10549-018-4982-9. Epub 2018 Sep 26. PMID: 30259284; PMCID: PMC6387648.
