# Supplementary material for: Identifying the effective behaviour change techniques in nutrition and physical activity interventions for the treatment of overweight/obesity in post-treatment breast cancer survivors: a systematic review
Source: Cancer Causes Control. 2023 May 6;34(8):683–703. doi: 10.1007/s10552-023-01707-w (PMC10267275; doi:10.1007/s10552-023-01707-w)
Supplement: Supplementary file 2 — Supplementary file2 (PDF 435 KB) [file 10552_2023_1707_MOESM2_ESM.pdf]

Identification of Theoretical Constructs and Intervention Techniques of the included studies.

Included Studies	Targeted Theoretical Constructs	Intervention Techniques Used
Rock, 2015 [40]	Self-efficacy<br>Self-management<br>Self-acceptance<br>Body image<br>Barriers	Goal-setting<br>Action planning<br>Self-monitoring<br>Problem solving<br>Relapse prevention<br>Role playing<br>Training
Demark-Wahnefried, 2014 [41]	Control of behavior<br>Positive reinforcement<br>Support<br>Barriers	Goal-setting<br>Action planning<br>Self-monitoring<br>Problem solving<br>Portion control<br>Feedback
Mefferd, 2006 [42]	Behavior and attitudinal change<br>Body image	Goal-setting<br>Self-monitoring<br>Cognitive restructuring<br>Monitor thoughts and feelings
Djuric, 2002 [43]	Self-regulatory processes<br>Self-efficacy<br>Self-acceptance<br>Positive thinking<br>Body image<br>Support<br>Barriers and environment cue elimination	Goal-setting<br>Action planning<br>Self-monitoring<br>Social eating
Sheppard, 2016 [44]	Attitudes<br>Subjective norms<br>Perceived control<br>Beliefs<br>Intentions<br>Body image<br>Self-efficacy<br>Barriers	Self-monitoring<br>Role modelling<br>Motivation

Included Studies	Targeted Theoretical Constructs	Intervention Techniques Used
Harrigan, 2016 [45]	Portion control<br>Cues<br>Coping with negative thoughts<br>Motivation	Goal-setting<br>Self-monitoring<br>Problem solving<br>Stimulus control<br>Relapse prevention<br>Feedback
Stolley, 2017 [46]	Self-efficacy<br>Health perspectives<br>Health promotion<br>Knowledge<br>Access to healthy eating resources<br>Tradition<br>Beliefs<br>Body image<br>Role expectation<br>Barriers<br>Support	Self-monitoring<br>Problem solving
Santa-Maria, 2020 [47]	Self-efficacy<br>Self-management<br>Optimism<br>Control<br>Support<br>Barriers	Goal-setting<br>Self-monitoring<br>Problem solving<br>Portion control<br>Time management<br>Stress management<br>Relapse prevention<br>Feedback
Reeves, 2017 [48]	Education<br>Advice<br>Promps<br>Motivation<br>Control<br>Support<br>Barriers	Goal-setting<br>Action planning<br>Self-monitoring<br>Problem solving<br>Stimulus control<br>Self-talk<br>Relapse prevention<br>Review<br>Reinforcement<br>Reward success

Included Studies	Targeted Theoretical Constructs	Intervention Techniques Used
Schmitz, 2019 [49]	Knowledge<br>Motivation<br>Negative thoughts<br>Barriers and cues<br>Body image<br>Support	Goal-setting<br>Self-monitoring<br>Problem solving<br>Practice<br>Prepare<br>Relapse prevention
Goodwin, 2014 [50]	Motivation<br>Negative thoughts<br>Barriers and social cues<br>Management<br>Self-esteem	Goal-setting<br>Self-monitoring<br>Problem solving<br>Time management<br>Distress<br>Relapse prevention<br>Review progress<br>Review problems

Theory Coding Scheme.

Studies	Rock, 2015 [40]	Demark-Wahnefried, 2014 [41]	Mefferd, 2006 [42]	Djuric, 2002 [43]	Sheppard, (2016) [44]	Harrigan, 2016 [45]	Stolley, 2017 [46]	Santa-Maria, 2020 [47]	Reeves, 2017 [48]	Schmitz, 2019 [49]	Goodwin, 2014 [50]
**1. Theory/ Model mentioned?**	Yes	Yes	Yes	Yes	Yes	Yes	Yes	Yes	Yes	Yes	DKC
**2. Targeted construct mentioned?**	Yes	Yes	Yes	Yes	Yes	Yes	Yes	Yes	Yes	Yes	Yes
**3. Single Theory?**	No	No	Yes	Yes	No	Yes	No	Yes	Yes	No	DK
**4. Theory/ predictors used to select recipients**	No	No	No	No	No	No	No	No	No	No	No

Studies	Rock, 2015 [40]	Demark-Wahnefried, 2014 [41]	Mefferd, 2006 [42]	Djuric, 2002 [43]	Sheppard, (2016) [44]	Harrigan, 2016 [45]	Stolley, 2017 [46]	Santa-Maria, 2020 [47]	Reeves, 2017 [48]	Schmitz, 2019 [49]	Goodwin, 2014 [50]
**5. Theory/pre-dictors used to select/develop IT.**	Yes	Yes	Yes	Yes	Yes	Yes	Yes	Yes	Yes	Yes	DK
**6. Theory/ predictors used to tailor IT to recipients**	Yes	Yes	Yes	Yes	Yes	Yes (protocol)	DK	DK	DK	DK	Yes
**7. All IT are explicitly linked to at least one theory-relevant construct/ predictor.**	Yes	Yes	Yes	Yes	Yes	Yes	Yes	Yes	Yes	Yes	Yes
**8. At least one but not all IT are explicitly linked to at least one theory-relevant construct/ predictor.**	Yes	Yes	Yes	Yes	Yes	Yes	Yes	Yes	Yes	Yes	Yes
**9. Group of techniques are linked to a group of constructs/ predictors.**	Yes	Yes	Yes	Yes	Yes	Yes	Yes	Yes	Yes	Yes	Yes
**10. All theory-relevant constructs/ predictors are explicitly linked to at least one IT.**	Yes	Yes	Yes	Yes	Yes	Yes	Yes	Yes	Yes	No	No

Studies	Rock, 2015 [40]	Demark-Wahnefried, 2014 [41]	Mefferd, 2006 [42]	Djuric, 2002 [43]	Sheppard, (2016) [44]	Harrigan, 2016 [45]	Stolley, 2017 [46]	Santa-Maria, 2020 [47]	Reeves, 2017 [48]	Schmitz, 2019 [49]	Goodwin, 2014 [50]
**11. At least one but not all of the theory-relevant constructs/ predictors are explicitly linked to at least one IT.**	Yes	Yes	Yes	Yes	Yes	Yes	Yes	Yes	Yes	Yes	Yes

IT = Intervention Technique. DK = Don’t Know.

Behaviour Change Techniques taxonomy mapping.

BCT’s	Rock, 2015 [40]	Demark-Wahnefried, 2014 [41]	Mefferd, 2006 [42]	Djuric, 2002 [43]	Sheppard, 2016 [44]	Harrigan, 2016 [45]	Stolley, 2017 [46]	Santa-Maria, 2020 [47]	Reeves, 2017 [48]	Schmitz, 2019 [49]	Goodwin, 2014 [50]	Promise Ratio
**1.1**	Y	Y	Y	Y	Y	Y	Y	Y	Y	Y	Y	10
**1.2**	Y	Y		Y	Y	Y	Y	Y	Y	Y	Y	9
**1.3**	Y			Y	Y	Y	Y	Y	Y	Y	Y	8
**1.4**	Y		Y	Y	Y	Y	Y		Y	Y		7
**1.5**	Y		Y	Y						Y	Y	5
**1.6**					Y					Y		1
**1.7**								Y	Y		Y	3
**2.2**	Y	Y	Y	Y		Y		Y	Y	Y	Y	9
**2.3**	Y	Y	Y	Y	Y	Y	Y	Y	Y	Y	Y	10
**2.4**	Y					Y	Y	Y	Y		Y	6

BCT’s	Rock, 2015 [40]	Demark-Wahnefried, 2014 [41]	Mefferd, 2006 [42]	Djuric, 2002 [43]	Sheppard, 2016 [44]	Harrigan, 2016 [45]	Stolley, 2017 [46]	Santa-Maria, 2020 [47]	Reeves, 2017 [48]	Schmitz, 2019 [49]	Goodwin, 2014 [50]	Promise Ratio
**2.6**									Y			1
**2.7**						Y		Y		Y	Y	4
**3.1**		Y	Y	Y							Y	4
**3.3**	Y				Y		Y	Y	Y	Y		5
**4.1**	Y	Y	Y	Y	Y	Y	Y	Y	Y	Y	Y	10
**5.1**			Y							Y	Y	3
**5.4**			Y									1
**6.1**	Y	Y			Y		Y	Y	Y	Y	Y	7
**6.2**								Y				1
**7.1**	Y		Y						Y	Y		4
**7.3**				Y								1
**8.1**	Y		Y				Y		Y	Y		5
**8.2**		Y					Y	Y	Y		Y	5
**8.3**									Y	Y		2
**8.4**		Y					Y	Y	Y		Y	5
**8.6**							Y	Y		Y		3
**8.7**	Y		Y		Y	Y	Y	Y	Y	Y	Y	8
**9.1**	Y	Y	Y	Y	Y	Y	Y	Y	Y	Y	Y	10

BCT's	Rock, 2015 [40]	Demark-Wahnefried, 2014 [41]	Mefferd, 2006 [42]	Djuric, 2002 [43]	Sheppard, 2016 [44]	Harrigan, 2016 [45]	Stolley, 2017 [46]	Santa-Maria, 2020 [47]	Reeves, 2017 [48]	Schmitz, 2019 [49]	Goodwin, 2014 [50]	Promise Ratio
**9.2**			Y				Y					2
**10.2**											Y	1
**10.4**									Y			1
**10.6**									Y			1
**10.9**									Y			1
**11.2**			Y		Y			Y		Y	Y	4
**12.1**	Y											1
**12.2**	Y							Y				2
**12.3**	Y			Y				Y			Y	4
**12.6**										Y		1
**13.1**					Y							0
**13.2**	Y		Y				Y					3
**13.4**			Y									1
**15.4**			Y		Y				Y			2
**Total**	19	10	18	12	14	11	17	20	23	21	20	

Inter-rater agreement for BTCs Taxonomy: Fleiss Multirater Kappa

Overall Agreement[a]

	Kappa	Asymptotic Standard Error	Asymptotic z	Asymptotic Sig.	Asymptotic 95% Confidence Interval Lower Bound	Asymptotic 95% Confidence Interval Upper Bound
Overall Agreement	.929	.018	51.454	<.001	.893	.964

a. Sample data contains 1023 effective subjects and 3 raters.

Agreement on Individual Categories[a]

Rating Category	Conditional Probability	Kappa	Asymptotic Standard Error	Asymptotic z	Asymptotic Sig.	Asymptotic 95% Confidence Interval Lower Bound	Asymptotic 95% Confidence Interval Upper Bound
0	.987	.929	.018	51.454	<.001	.893	.964
1	.942	.929	.018	51.454	<.001	.893	.964

a. Sample data contains 1023 effective subjects and 3 raters.

Inter-rater agreement for Interventions' Promise Classification: Crosstabs

Symmetric Measures

		Value	Asymptotic Standard Error[a]	Approximate T[b]	Approximate Significance
Measure of Agreement	Kappa	.593	.244	2.884	.004
N of Valid Cases		11			

a. Not assuming the null hypothesis.

b. Using the asymptotic standard error assuming the null hypothesis.

Studies	TIDier Checklist (BRIEF NAME; WHY; WHAT; WHO PROVIDED; HOW; WHERE; WHEN AND HOW MUCH; TAILORING; MODIFICATIONS; HOW WELL (Planned), and HOW WELL (Actual).
**Rock, 2015 [40]**	**Brief Name:** Exercise and Nutrition to Enhance Recovery and Good Health for you (ENERGY) Trial (p3169, 2015).<br>**WHY:** To determine whether a behavioral WL intervention emphasizing increased PA and tailored to BCS would result in greater WL in the IG compared with a CG assigned to a less intensive intervention (p3170, 2015).<br>**Theory:** SCT and Cognitive - Behavioural treatment of obesity with MIT (p286, 2013).<br>**WHAT:** Materials: Tailored print materials were adapted from the Fresh Start (doi: 10.1249/01.MSS.0000053704.28156.0F) and RENEW (doi: 10.1002/pon.1491) trials with web-based resources, digital videos, pedometers and weight records. Procedures: The goal was a modest WL of at least 7% BW through behavioral goals such as reduced energy intake and increased physical activity, by personalized guidance (p286-7, 2013). Detailed information can be found in<br>.<br>**WHO:** leaders, who had backgrounds in dietetics, psychology and/or exercise physiology (p286, 2013).<br>**HOW:** face-to-face group sessions, emails, telephone, newsletters (p286-7, 2013).<br>**WHERE:** USA (University of California, San Diego [UCSD]; University of Colorado Denver; University of Alabama at Birmingham; and Washington University in St. Louis [WUSTL]) (p.284, 2013).<br>**WHEN AND HOW MUCH:** 6 months intensive phase: 4 months, 1h weekly group sessions and next 2 months, 1h group sessions every other week. 6-12 months: monthly meetings, followed by brief (10-15 min) personalised guidance delivered by tel and/or email. 6-24 months: tailored newsletters. Physical Activity: Step-wise increase to 60 min/day of moderate intensity purposeful exercise, 10000 steps per day & 2-3 times/week strength training at home or an exercise facility (p.286-7, 2013).<br>**TAILORING:** individualize the feedback, goal-setting, planning and follow-through for the behavioral goals, diet and PA (p.286-7, 2013).<br>**MODIFICATIONS:** None described.<br>**HOW WELL (planned):** quality assurance and monitoring is described in detail (p.290, 2013).<br>**HOW WELL (actual):** 12.8% (N=44/344) did not complete the study (p.3170, 2015).

Studies	TIDier Checklist (BRIEF NAME; WHY; WHAT; WHO PROVIDED; HOW; WHERE; WHEN AND HOW MUCH; TAILORING; MODIFICATIONS; HOW WELL (Planned), and HOW WELL (Actual).
**Demark-Wahnefried, 2014 [41]**	**Brief Name:** Daughters and Mothers Against Breast Cancer (DAMES) trial (p2522).<br>**WHY:** endeavored to capitalize on the mother-daughter bond and the teachable moment created by a cancer diagnosis to promote weight loss in overweight or obese women recently diagnosed with breast cancer and their overweight or obese daughters (p2523).<br>**Theory:** SCT & Transtheoretical model of behaviour change, plus concepts of independence theory and the theory of communal coping (p2526).<br>**WHAT:** Materials: workbook that was personalized with reinforced goals proposed by the ACS and the US dietary guidelines, 6 newsletters, logbooks, reference manuals, web sites, portion control tableware, iPods, shoes chips. Procedures: promoted portion control and diets high in nutrients and low in energy as well as 150 minutes per week of aerobic exercise and twice-weekly strength training (p2526-7).<br>**WHO:** Not described.<br>**HOW:** emails and newsletters (p2526).<br>**WHERE:** USA, Puerto Pico or Guam (through the cancer registries of Duke University and UT-MDACC and regions in which there was visiting nurse coverage by Examination Management Services Inc) (p.2524-5).<br>**WHEN AND HOW MUCH:** 6 months intervention and 6 months follow-up phase (p.2523-4).<br>**TAILORING:** interventions differed with respect to tailoring (p.2526).<br>**MODIFICATIONS:** None described.<br>**HOW WELL (planned):** Preestablished benchmarks were the achievement of: 1) targeted accrual within a 9-month period; 2) "good" adherence as noted by the completion of at least 4 of 6 of the written surveys; 3) a retention rate of at least 80%; and 4) an absence of serious adverse events that were directly attributable to the intervention (p.2527).<br>**HOW WELL (actual):** 66% adherence (in dyads intervention group) as noted by the completion of at least 4 of 6 of the written surveys and a retention rate of at least 90% (p2524) and more details in adhering and retention (p2528).
**Mefferd, 2007 [42]**	**Brief Name:** The Healthy Weight Management (HWM) Study (p146, 2007).<br>**WHY:** The intervention incorporated CBT, emphasizing PA, diet modification to facilitate a modest reduction in energy intake, and strategies to improve body image and self-acceptance (p146, 2007).<br>**Theory:** Cognitive Behavioural Therapy (p146, 2007).<br>**WHAT:** Materials: food diaries, exercise logs, pedometers and intervention material. Procedures: 500-1000 kcal deficit for a WL by increasing high-fibre vegetables, whole grains & fruits and regular aerobic exercise with a step-wise increase in time and intensity (1h/day of moderate to vigorous PA & 2-3 times/week muscle strengthening) (p147, 2007).<br>**WHO:** trained investigators and research staff (p.335, 2011, DOI 10.1007/s12529-010-9079-8).<br>**HOW:** in-person group sessions and telephone contacts (p147, 2007).<br>**WHERE:** USA (San Diego) (p.334, 2011, DOI 10.1007/s12529-010-9079-8).<br>**WHEN AND HOW MUCH:** 16 weeks of weekly closed groups sessions followed by once-monthly session till 12 months. (p.146, 2007).<br>**TAILORING:** Individualized telephone counseling to individualize goal-setting and assess progress (p.146, 2007).<br>**MODIFICATIONS:** None described.<br>**HOW WELL (planned):** Not described.<br>**HOW WELL (actual):** Of the participants in the intervention group, attendance at the weekly sessions was 80% on average and a retention rate of almost 84% (p148, 2007).

Studies	**TIDier Checklist** (BRIEF NAME; WHY; WHAT; WHO PROVIDED; HOW; WHERE; WHEN AND HOW MUCH; TAILORING; MODIFICATIONS; HOW WELL (Planned), and HOW WELL (Actual)**.**
**Djuric, 2002 [43]**	**Brief Name:** Randomized pilot study tested an individualized approach toward weight loss in obese Breast cancer survivors (p657).<br>**WHY:** individualized counseling methods typically have not been used in WL research studies but this approach is sensitive to the needs and abilities of each individual (p658).<br>**Theory:** SCT (p659).<br>**WHAT:** Materials: A monthly packet of written information on various WL topics (environmental control, serving-size control, exercise, motivation, goal setting, holiday eating, seasonal foods), pedometers, exercise and dietary logs. Procedures: 10% WL of BW through 500-1000 kcal deficit for a WL of 1-2 pounds/week, by decreasing energy & fat & increasing fibres and at least 5 servings/day fruits & vegetables plus 30-45 min/day moderate activity most days of the week. (p659).<br>**WHO:** Registered dietian (p.659).<br>**HOW:** in-person group meetings, telephone individual counselling and emails (p659).<br>**WHERE:** USA.<br>**WHEN AND HOW MUCH:** weekly sessions for the first 3 months, biweekly for 3-6 months & monthly thereafter (p.659).<br>**TAILORING:** depending on individual needs (p.659).<br>**MODIFICATIONS:** None described.<br>**HOW WELL (planned):** Not described.<br>**HOW WELL (actual):** From 0 to 3 months or from 0 to 6 months, the associations of weight loss with attendance at these groups was not statistically significant, but from 0 to 12 months was significant (r=0.775, p=0.014), with subjects attending 9% to 92% of the monthly groups and 28% or more of the sessions, in 12 months (p661-2).
**Sheppard, 2016 [44]**	**Brief Name:** Stepping Stone (Survivors Taking on Nutrition and Exercise) study (p107).<br>**WHY:** Studies with white survivors suggest that interventions are more effective when they are multifaceted, personalized, teach behavioral skills, provide social support, and increase self- efficacy. This is also likely true for black survivors, but documentation of successful strategies for them are lacking (p106-7).<br>**Theory:** Social Cognitive Theory (SCT) & Theory of Planned Behaviour (TPB), with Motivational Interviewing (p108).<br>**WHAT:** Materials: pedometers, notebooks, tools to monitor and track their daily food intake, and binders to store resources and session materials. Procedures: WL of at least 5% BW in 12 weeks, through 1 pound of WL per week, >5 fruits and vegetables/day and <35% kcal from total fat and moderate intensity exercise of >30 min/day, ≥ 5 days/week, and 10000 steps/day (p108).<br>**WHO:** exercise physiologist, nutritionist and trained survivor coach (p108).<br>**HOW:** in-person group sessions, plus individual telephone coaching sessions (p108).<br>**WHERE:** USA.<br>**WHEN AND HOW MUCH:** 12 weeks intervention: once biweekly a 90-min group sessions (30 min PA & 60 min education sessions), plus individual telephone (15 min) coaching sessions every other week (p.108).<br>**TAILORING:** individualized sessions were tailored to baseline intentions, attitudes, and subjective norms (p.108).<br>**MODIFICATIONS:** None described.<br>**HOW WELL (planned):** Interventionists (i.e., nutritionists, exercise physiologists, survivor-coaches) received a detailed intervention-manual (included session objectives, tasks, scripts, activities, and resources), an overview of breast cancer and training in MIT (p.107-8). Satisfaction with interventionists was assessed with Likert-formatted items (p.109).<br>**HOW WELL (actual):** 67% completed the study (in the intervention group) (p.109).

Studies	TIDier Checklist (BRIEF NAME; WHY; WHAT; WHO PROVIDED; HOW; WHERE; WHEN AND HOW MUCH; TAILORING; MODIFICATIONS; HOW WELL (Planned), and HOW WELL (Actual).
**Harrigan, 2016 [45]**	**Brief Name:** The Lifestyle, Exercise, and Nutrition (LEAN) Study (p669).<br>**WHY:** Telephone-based weight loss counseling may be a viable time-effective alternative to in-person visits (p670).<br>**Theory:** Social Cognitive Theory (SCT) (p670).<br>**WHAT:** Materials: 11-chapter LEAN book, daily record of all food and beverage intake, minutes of physical activity, and pedometer steps in the LEAN Journal and weighed themselves once per week with a scale (HoMedics), and recorded their weight in the LEAN Journal. Procedures: WL of at least 10% BW in 6 months, through 500 kcal energy deficit based on a plant-based diet reducing sugars and increasing fibre and home-based PA with a goal of 150 min per week moderate-intensity activity, such as brisk walking, with a daily target of 10000 steps (p670-1).<br>**WHO:** RD specialised in oncology nutrition and trained in exercise physiology & behaviour modification counselling (p670).<br>**HOW:** either in-person or telephone individual sessions (p670).<br>**WHERE:** Yale, USA (p.670).<br>**WHEN AND HOW MUCH:** 6 months, 11 sessions (30-min counselling), (once weekly the first month, once biweekly the second & third month and once monthly for the months 4,5,6) (p.670).<br>**TAILORING:** participants received individualized counseling sessions (p.670).<br>**MODIFICATIONS:** None described.<br>**HOW WELL (planned):** described in detail in appendix (protocol information) (p.14-15, protocol).<br>**HOW WELL (actual):** A total of 61% and 47% of women randomly assigned to in-person and telephone counseling, respectively, participated in all 11 counseling sessions, and 91% and 71%, respectively, completed the study (p.671-3).
**Stolley, 2017 [46]**	**Brief Name:** Moving Forward trial, a WL intervention for African American BCS on weight, body composition and behavior (p2820, 2017).<br>**WHY:** Body composition and biological data will enhance the understanding of how WL may impact BC recurrence risk and overall health risk among African-American women (p2, 2015, study design, DOI 10.1186/s12885-015-2004-4. Study conceptual framework in fig.2, page 3).<br>**Theory:** Social Cognitive Theory (SCT) and Socio-Ecological Model (SEM), with Motivational Interviewing (p6, 2015, study design).<br>**WHAT:** Materials: classes with specific topics of diet and exercise, weight, food and activity records, program binder with handouts, recipes, and other supportive materials (p2822, 2017 and p7, 2015 study design). Procedures: WL of at least 5% BW in 6 months, through 500 kcal deficit by increasing fruit & vegetable consumption and PA ≥ 150 min per week (p2821, 2017).<br>**WHO:** a community dietitian, a community cancer exercise instructor, and a health psychologist (p7, 2015, study design).<br>**HOW:** in-person group sessions, text messages through a software application, mytapp and newsletters (p7-8, 2015, study design).<br>**WHERE:** USA, Chicago area (p.2821, 2017).<br>**WHEN AND HOW MUCH:** twice - weekly (for **26 weeks**) in-person classes with supervised exercise & text messaging (p.2822, 2017).<br>**TAILORING:** Not described.<br>**MODIFICATIONS:** Intervention goals change briefly (p.6, 2015, study design vs p2821, 2017).<br>**HOW WELL (planned):** Not described.<br>**HOW WELL (actual):** Participants attended an average of 55% of the 48 classes offered (p.2823, 2017). 88,8% complete the 6-months intervention and 85,6% the 12-months follow-up (p.2821, 2017).

Studies	**TIDier Checklist** (BRIEF NAME; WHY; WHAT; WHO PROVIDED; HOW; WHERE; WHEN AND HOW MUCH; TAILORING; MODIFICATIONS; HOW WELL (Planned), and HOW WELL (Actual)**.**
**Santa-Maria, 2020 [47]**	**Brief Name:** POWER-remote trial, Practice-based Opportunities for Weight Reduction for breast cancer survivors (p3024-5, 2020).<br>**WHY:** BC Patients with obesity experience inferior outcomes, biologically related to metabolic and inflammatory pathways, and other molecular changes. WL may be associated with decreases in leptin and other inflammatory markers, which may have antioncogenic effects (p3025, 2020).<br>**Theory:** Social Cognitive Theory with Motivational Interviewing (p3025, 2020).<br>**WHAT:** Materials: educational materials included oncology-relevant information such as lymphedema prevention exercises and general information about BC, web-based resources with objectives, educational content, quizzes, and supporting worksheet and self-monitoring tools and graphs (weight, minutes of exercise/day, calories consumed/day). Procedures: WL of at least 5% BW in 6 months, through 1200-2200 kcal/day energy intake depending on BW based on DASH dietary pattern: 7-12 servings of fruits/vegetables, 2-3 servings of low fat dairy, reduced sodium & ≤ 25% of calories from fat and built up to ≥ 300 min/week of moderate intensity PA in bouts ≥ 10 min in length (p3025, 2020 and supplemental material, table 1, more information can be found in supplementary appendix 1, paper 2011, doi:10.1056/NEJMoa1108660).<br>**WHO:** health coaches with a background in delivering weight loss interventions (p3025, 2020).<br>**HOW:** telephone-based behavioral WL coaching and use of a web-based self-monitoring and learning platform (p3025, 2020).<br>**WHERE:** USA.<br>**WHEN AND HOW MUCH:** 12-months (telephone-based coaching & use of a web-based self-monitoring and learning platform). A total of 21 phone calls: weekly for 3 months & monthly for 9 months (20 min calls per session). (p3025, 2020).<br>**TAILORING:** Individually tailored (supplemental material, table 1, 2020).<br>**MODIFICATIONS:** None described.<br>**HOW WELL (planned):** Coaches trained in both behavioral WL principles and MI (p3025, 2020) and details about sample size (p3026, 2020).<br>**HOW WELL (actual):** High participation rates, median call completion was 14/15 calls in the first 6 months and 7/7 calls from months 7–12. There was a median of 24 weekly logins during the first 6 months and 22.5 weekly logins during months 7–12 (p3027, 2020). Only one patient randomized to POWER-remote group was lost to follow up (p3028, 2020).

Studies	**TIDier Checklist** (BRIEF NAME; WHY; WHAT; WHO PROVIDED; HOW; WHERE; WHEN AND HOW MUCH; TAILORING; MODIFICATIONS; HOW WELL (Planned), and HOW WELL (Actual)**.**
**Reeves, 2017 [48]**	**Brief Name:** Living Well after Breast Cancer Pilot Trial (p125, 2017).<br>**WHY:** Comparisons of interventions against usual care are still warranted, particularly when examining patient-reported outcomes and treatment-related side-effects, as these may naturally improve over time following treatment completion (p2, 2016, study protocol, DOI 10.1186/s12885-016-2858-0).<br>**Theory:** Social Cognitive Theory with Motivational Interviewing (p127, 2017).<br>**WHAT:** Materials: a detailed workbook, self-monitoring diary, digital scales, pedometer, calorie-counter book, food model booklet. Procedures: WL of 5-10% BW in 6 months, through 2000 kj (≈ 500 kcal) daily energy deficit aiming to ≤30% total fat, <7% saturated fat, 5 servings/day vegetables, 2 servings/day fruits, limit alcohol intake & portion control and gradually increased moderate intensity planned PA to at least 30-min/day (≥ 210 min/week) and 10000 steps/daily (p127, 2017 and p4, and 10, 2016, study protocol).<br>**WHO:** lifestyle coaches, who were accredited practicing dietitians trained in exercise promotion and motivational interviewing (p127, 2017).<br>**HOW:** individual telephone sessions, optional supportive text messages and newsletters (p5, 2016, study protocol).<br>**WHERE:** Australia, within 50 km of the state capital, Brisbane (p126, 2017).<br>**WHEN AND HOW MUCH:** 12 months: 6 months initial phase: A total of 16 phone calls (weekly for 6 weeks & 10 fortnightly calls) and 6 months extended care phase: 6 monthly calls (p5, 2016, study protocol).<br>**TAILORING:** tailored to the participant's preferences (p6, 2016, study protocol) and individualized guidance (p127, 2017).<br>**MODIFICATIONS:** None described.<br>**HOW WELL (planned):** Call checklists were used by coaches to facilitate intervention fidelity (p127, 2017) and retention strategies in details (p11, 2016, study protocol).<br>**HOW WELL (actual):** 69% of participants received ≥75% possible calls, and 11,1% was lost to follow up (p128, 2017), details about acceptability and intervention implementation and satisfactions (p131, 2017).

Studies	**TIDier Checklist** (BRIEF NAME; WHY; WHAT; WHO PROVIDED; HOW; WHERE; WHEN AND HOW MUCH; TAILORING; MODIFICATIONS; HOW WELL (Planned), and HOW WELL (Actual)**.**
**Schmitz, 2019 [49]**	**Brief Name:** Women in Steady Exercise Research (WISER) Survivor clinical trial (p1605, 2019).<br>**WHY:** to test the effects of exercise and/or WL on lymphedema, biomarkers for recurrence and quality of life. The hypothesis is that exercise and weight loss will affect these outcomes, but that the combined effect will be larger (p64, 2017, study design, doi:10.1016/j.cct.2017.07.017).<br>**Theory:** Social Cognitive Theory and Behavioral Self-Management Theory, with Motivational Interviewing (p17, appendix - protocol of POWER-UP trial, 2011, DOI: 10.1056/NEJMoa1109220).<br>**WHAT:** Materials: exercise and food logs using an electronic food diary accessible through the WISER Survivor website (p12-13, supplement 1, study protocol, 2019). Procedures: WL of 10% BW in 6 months, based on the guidelines from ACS along with a meal replacement program (Nutrisystem) & 7 servings fruits and vegetables daily, plus twice per week resistance exercise per 90-min class along with aerobic activity to 180 min per week (p11-13, supplement 1, study protocol, 2019).<br>**WHO:** registered dietitians experienced with the NutriSystem program and exercise by certified exercise instructors (p11-12, supplement 1, study protocol, 2019).<br>**HOW:** in-person group meetings along with telephone individual counselling (p11-12, supplement 1, study protocol, 2019).<br>**WHERE:** USA, Pennsylvania (p5, supplement 1, study protocol, 2019).<br>**WHEN AND HOW MUCH:** 12 months (52 weeks) home-based exercise program of strength training twice/week & 180 min/week walking along with 24 weeks nutritional counseling group sessions (p11-13, supplement 1, study protocol, 2019).<br>**TAILORING:** will develop a tailored diet that meets the same calorie control goals (p12, supplement 1, study protocol, 2019) and the WL intervention was tailored to the needs of breast cancer survivors (p66, 2017, study design)<br>**MODIFICATIONS:** None described.<br>**HOW WELL (planned):** Participant adherence will be monitored by intervention staff on an ongoing basis. Participant adherence to the exercise and WL protocols will be determined based on weekly review of exercise training log data, review of food logs at each in-person weight-loss intervention meeting, and weigh-ins at each in-person WL meeting (p11, supplement 1, study protocol, 2019 and p67, 2017, study design).<br>**HOW WELL (actual):** Women randomized to the exercise and combined groups completed an average of 72 of the 100 prescribed home-based re- sistance exercise sessions (72%) and an average of 133 of the 180 prescribed aerobic exercise minutes per week (74%) (p1610, 2019) and participants retention in the combined group was at 84% (p1607, 2019).

Studies	**TIDier Checklist** (BRIEF NAME; WHY; WHAT; WHO PROVIDED; HOW; WHERE; WHEN AND HOW MUCH; TAILORING; MODIFICATIONS; HOW WELL (Planned), and HOW WELL (Actual)**.**
**Goodwin, 2014 [50]**	**Brief Name:** Lifestyle Intervention in Adjuvant Treatment of Early Breast Cancer (LISA) trial (p2231, 2014).<br>**WHY:** Obesity is a complex physiologic state associated with insulin resistance, higher levels of circulating insulin, an altered adipocytokine profile (increased leptin, decreased adiponectin), and generalized inflammation. WL may improve BC outcomes (p2231-2, 2014).<br>**Theory:** Social Cognitive Theory with Motivational Interviewing (p1, 2020, and DPP intervention manual at).<br>**WHAT:** Materials: Detailed patient workbook which focus on weight control through healthy diet and exercise, with logs and pedometer (p18, protocol, supplement, 2014). Procedures: WL of 10% BW in 6 months, through 500-1000 kcal deficit for a WL of 1-2 lbs/week, by decreasing fat to 20% of total intake & increasing fruits, vegetables & fibres and a gradual increase in moderate-intensity aerobic physical activity (walking for the majority of participants) to 150 to 200 minutes per week (p2233, 2014).<br>**WHO:** trained lifestyle coaches (p2233, 2014).<br>**HOW:** individual telephone sessions and newsletters (p2233, 2014).<br>**WHERE:** Canada, Ontario Clinical Oncology Group (p2232, 2014).<br>**WHEN AND HOW MUCH:** 24 months telephone-based intervention: 6 months of the intensive (weekly for 4 weeks) & consolidation phase (fortnightly for 2-6 months) & 18 months of the maintenance phase (every 2 months for 7-12 and every 3 months for 12-24 months) (p2233, 2014).<br>**TAILORING:** Lifestyle coaches individualized the intervention as necessary (p2233, 2014).<br>**MODIFICATIONS:** Patients with N3 tumor characteristic were allowed in initial protocol, amended June 2008, when 49 patients had been accrued (p2234, 2014).<br>**HOW WELL (planned):** described in details in 5.5.2.2 standardization of the intervention (p28-30, protocol, supplement, 2014).<br>**HOW WELL (actual):** 95.9% received at least one phone call, and 2,636 total calls were completed (81.1% of those specified by the protocol). 62% completed all 19 calls (p2234, 2014) and participants retention was 94% in 6 months, 83% in 12months, 79% in 18months and 76% in 24months follow up (p2232 and p2238, 2014).
