# Supplementary material for: Identifying the effective behaviour change techniques in nutrition and physical activity interventions for the treatment of overweight/obesity in post-treatment breast cancer survivors: a systematic review
Source: Cancer Causes Control. 2023 May 6;34(8):683–703. doi: 10.1007/s10552-023-01707-w (PMC10267275; doi:10.1007/s10552-023-01707-w)
Supplement: Supplementary file 3 — Supplementary file3 (PDF 162 KB) [file 10552_2023_1707_MOESM3_ESM.pdf]

Analysis of the Rob-2 scoring.

Domains		Rock, 2015 [40]	Demark-Wahne-fried, 2014 [41]	Mefferd, 2006 [42]	Djuric, 2002 [43]	Sheppard, 2016 [44]	Harrigan, 2016 [45]	Stolley, 2017 [46]	Santa-Maria, 2020 [47]	Reeves, 2017 [48]	Schmitz, 2019 [49]	Goodwin, 2014 [50]
**D1**	1.1	Y	PY	PY	PY	NI	Y	Y	Y	Y	Y	Y
	1.2	PY	Y	NI	PY	NI	Y	Y	PY	Y	Y	Y
	1.3	N	N	N	PN	PN	N	PN	N	N	N	PN
	**D1 Total**	**LOW**	**LOW**	**SC**	**LOW**	**SC**	**LOW**	**LOW**	**LOW**	**LOW**	**LOW**	**LOW**
**D2**	2.1	PY	N	PY	PY	PY	PY	PY	Y	PY	PY	PY
	2.2	PY	Y	PY	PY	PY	PY	PY	PY	PY	PY	PY
	2.3	NI	NI	NI	NI	NI	NI	NI	NI	NI	NI	NI
	2.6	PN	PY	PN	NI	PY	Y	PY	Y	PY	Y	Y
	2.7	PN		PY	PY							
	**D2 Total**	**SC**	**SC**	**HIGH**	**HIGH**	**SC**	**SC**	**SC**	**SC**	**SC**	**SC**	**SC**
**D3**	3.1	PY	Y	N	PN	N	PY	PN	PY	PY	PY	Y
	3.2			PN	PN	PY		PN				
	3.3			PN	PN			PN				
	**D3 Total**	**LOW**	**LOW**	**LOW**	**LOW**	**LOW**	**LOW**	**LOW**	**LOW**	**LOW**	**LOW**	**LOW**
**D4**	4.1	N	N	PN	N	NI	N	N	PN	N	PN	N
	4.2	N	N	N	N	N	N	N	N	N	N	N
	4.3	PN	PY	PY	NI	PY	PY	PY	N	N	N	PY

Domains		Rock, 2015 [40]	Demark-Wahne-fried, 2014 [41]	Mefferd, 2006 [42]	Djuric, 2002 [43]	Sheppard, 2016 [44]	Harrigan, 2016 [45]	Stolley, 2017 [46]	Santa-Maria, 2020 [47]	Reeves, 2017 [48]	Schmitz, 2019 [49]	Goodwin, 2014 [50]
	4.4		PN	PN	PN	PN	PN	PN				PN
	**D4 Total**	**LOW**	**LOW**	**LOW**	**LOW**	**LOW**	**LOW**	**LOW**	**LOW**	**LOW**	**LOW**	**LOW**
**D5**	5.1	Y	Y	NI	NI	PY	PY	Y	PY	Y	Y	PY
	5.2	N	N	N	N	N	N	N	N	N	N	N
	5.3	N	PN	NI	NI	NI	N	PN	PN	PN	PN	PN
	**D5 Total**	**LOW**	**LOW**	**SC**	**SC**	**SC**	**LOW**	**LOW**	**LOW**	**LOW**	**LOW**	**LOW**
**Overall**		**SC**	**SC**	**HIGH**	**HIGH**	**HIGH**	**SC**	**SC**	**SC**	**SC**	**SC**	**SC**

Y = yes, PY = probably yes, N = no, PN = probably no, NI = no information, SC = some concerns.

Crosstabs

Symmetric Measures

		Value	Asymptotic Standard Error[a]	Approximate T[b]	Approximate Significance
Measure of Agreement	Kappa	.694	.030	24.893	<.001
N of Valid Cases		308			

a. Not assuming the null hypothesis.

b. Using the asymptotic standard error assuming the null hypothesis.
